# Spatial Variation of the Microbial Community Structure of On-Site Soil Treatment Units in a Temperate Climate, and the Role of Pre-treatment of Domestic Effluent in the Development of the Biomat Community

**DOI:** 10.3389/fmicb.2022.915856

**Published:** 2022-06-24

**Authors:** Alejandro Javier Criado Monleon, Jan Knappe, Celia Somlai, Carolina Ospina Betancourth, Muhammad Ali, Thomas P. Curtis, Laurence William Gill

**Affiliations:** ^1^Department of Civil, Structural and Environmental Engineering, Trinity College Dublin, The University of Dublin College Green, Dublin, Ireland; ^2^Mathematics Applications Consortium for Science and Industry (MASCI), Limerick University, Limerick, Ireland; ^3^Department of Engineering, Newcastle University, Newcastle upon Tyne, United Kingdom; ^4^Biological and Environmental Science and Engineering Division, Water Desalination and Reuse Research Center, King Abdullah University of Science and Technology, Thuwal, Saudi Arabia

**Keywords:** bioclogging, on-site wastewater treatment, soil treatment unit, microbial diversity, microbial community structure, microbial community composition

## Abstract

The growth of microbial mats or “*biomats*” has been identified as an essential component in the attenuation of pollutants within the soil treatment unit (STU) of conventional on-site wastewater treatment systems (OWTSs). This study aimed to characterize the microbial community which colonizes these niches and to determine the influence of the pre-treatment of raw-domestic wastewater on these communities. This was achieved through a detailed sampling campaign of two OWTSs. At each site, the STU areas were split whereby half received effluent directly from septic tanks, and half received more highly treated effluents from packaged aerobic treatment systems [a coconut husk media filter on one site, and a rotating biodisc contactor (RBC) on the other site]. Effluents from the RBC had a higher level of pre-treatment [~90% Total Organic Carbon (TOC) removal], compared to the media filter (~60% TOC removal). A total of 92 samples were obtained from both STU locations and characterized by 16S rRNA gene sequencing analysis. The fully treated effluent from the RBC resulted in greater microbial community richness and diversity within the STUs compared to the STUs receiving partially treated effluents. The microbial community structure found within the STU receiving fully treated effluents was significantly different from its septic tank, primary effluent counterpart. Moreover, the distance along each STU appears to have a greater impact on the community structure than the depth in each STU. Our findings highlight the spatial variability of diversity, Phylum- and Genus-level taxa, and functional groups within the STUs, which supports the assumption that specialized biomes develop around the application of effluents under different degrees of treatment and distance from the source. This research indicates that the application of pre-treated effluents infers significant changes in the microbial community structure, which in turn has important implications for the functionality of the STU, and consequently the potential risks to public health and the environment.

**Graphical Abstract:**
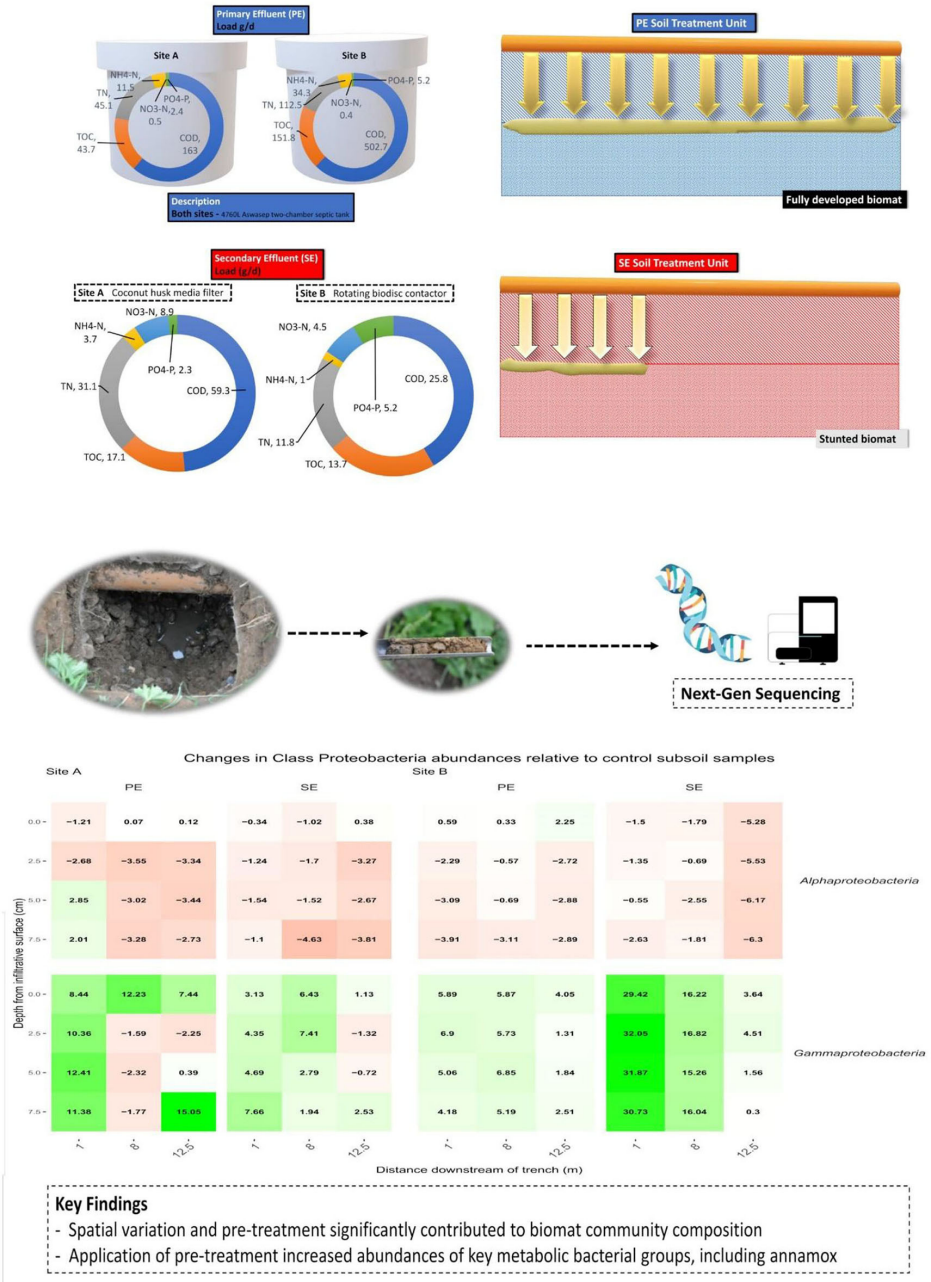
Influence of pre-treatment on the microbial community structure and biomat development.

## Introduction

A typical on-site wastewater treatment system (OWTS) uses a septic tank to provide primary treatment and a limited amount of anaerobic digestion. Further treatment occurs as effluent percolates through the soil-stone matrix of the soil treatment unit (STU) which can vary in their configuration according to site specific design requirements (Gill, 2011). The underlying soil or subsoil into which the wastewater effluent percolates provides a critical buffer zone for the protection of water resources (Amador and Loomis, 2019). The key to effective on-site treatment is to maintain an unsaturated subsoil through which the effluent can percolate freely, wherein chemical and microbiological contaminants will be attenuated to an acceptable level before they reach the groundwater (Siegrist, 2017). In Ireland, for example, the Environmental Protection Agency's Code of Practice dictates that the septic tank effluent requires a minimum of 1.2 m unsaturated subsoil depth below the invert of the percolation trenches to the water table and/or bedrock with a 18-m long percolation trench required per household occupant (EPA, 2021). Similar design criteria are used in other countries such as the USA, Canada, Australia, and UK.

More recently, there has been a proliferation of packaged treatment systems that provide additional (secondary) treatment to the effluent before being discharged to the STU. Regardless of using a secondary treatment unit, the STU remains a crucial component of domestic wastewater treatment, particularly, the development of the biomat or “clogging layer,” which grows over the base of the STUs correlated to the level of organic and nutrient loading (Bouma, 1975; Siegrist and Boyle, 1987). This layer causes a sharp drop in infiltration rates, initially due to physical clogging processes, followed by more gradual clogging over several months resulting from the development of the microbial biomat formed by the production of extracellular polymeric substances (EPS) (Beal et al., 2005). Studies have linked the effects of the organic loading rates on the biomat development, comparing the trenches dosed with primary (septic tank) effluents and secondary treated effluents (Gill et al., 2007; Knappe et al., 2020). These studies also showed that the nitrogen loading at the base of the STUs receiving secondary effluents was 2–3 times higher than that in percolation areas receiving septic tank effluents (Gill et al., 2009).

Most on-site wastewater treatment studies have focused on the system performance for the attenuation of hazardous contaminants from an environmental and public health perspective. These studies have generally focused on chemical parameters such as nutrients (different forms of nitrogen and phosphorus), bulk organics (BOD, COD, and TOC), and fecal indicator bacteria such as *E. coli*, enterococci, and bacteriophages as surrogates for human enteric viruses (van Cuyk and Siegrist, 2007; Gill et al., 2009; O'Luanaigh et al., 2012; Humphrey Jr et al., 2019). Increasingly, since the advance of microbiological culture-independent techniques in the early 1990s (Wagner et al., 2006), the performance of wastewater treatment systems has been coupled with the dynamics and stochastic modeling of the composition of microbial communities (Curtis and Sloan, 2005; Sanz and Köchling, 2007; Siezen and Galardini, 2008; Matar et al., 2021). To date, nitrogen removal processes (ammonia oxidation, nitrite oxidation, denitrification, and anammox), phosphorous removal, floc, and biofilm formation (Daims et al., 2006) have been highly focused on centralized wastewater treatment processes. With advances in sequencing technology and expansions in genomic databases, more detailed microbial community profiles have been developed for suspended growth flocs (in activated sludge), attached growth fixed-film treatment systems (trickling filters, etc.) (Sanz and Köchling, 2007), and suspended growth biofilm treatment systems (Ali et al., 2019).

Several factors impact the microbial community structure within a wastewater treatment system, including influent composition, environmental conditions, system processes, plant configuration, and operational parameters (Hu et al., 2012; Lee et al., 2015; Chen et al., 2017; Zhang et al., 2020). The propagation of cheaper and more accurate next-generation sequencing has allowed for a greater resolution of community structure, deeper sequencing, and thus a better determination of the processes behind community assembly, such as dispersal (immigration from the influent), selection (deterministic, driven by taxa fitness and environmental factors), and ecological drift (temporal changes in abundances caused by stochastic events) (Ali et al., 2019; Frigon and Wells, 2019; Dottorini et al., 2021).

Many of the studies on microbiological analysis associated with wastewater treatment processes have been confined to centralized wastewater systems, as highlighted above. Within on-site soil filtration systems, Tomaras et al. (2009) presented one of the first sequencing profiles of soil microorganisms from the biomat of the STU, finding that microbes found within septic tank effluents were absent in the biomat. Depth has also been considered as a contributor to the microbial community structure (Truu et al., 2009). Effluent storage has shown to cause reductions in microbial diversity as high nutrient contents are gradually degraded (Knisz et al., 2021) with comparisons of the effluent from multiple on-site systems showing the effect of treatment technologies and seasonality on the structure of nitrifying and denitrifying communities (Ross et al., 2020). Crucially, studies have reported that, in contrast to centralized wastewater treatment plants (WWTPs), OWTSs appear to be highly influenced by the inoculation of soil organisms during installation and maintenance (Wigginton et al., 2020). However, the microbial community structure of STUs is understudied. This lacuna is likely due to the laborious and time-consuming nature of sampling within the soil system. In this study, we presented a high-resolution profile of the community structure across two separate OWTSs with relatively similar subsoil and land use that enables a more direct comparison of the effect of treated effluent dispersal on the STU biome. This study expands the existing knowledge on STU biomat growth by providing valuable insights into the community structure across the length and depth of several systems, each receiving an effluent with varying levels of pre-treatment. The findings of this study also have implications in other related fields such as reclaimed water irrigation and groundwater recharge.

## Methods

### Site Description

**Figure 1:**
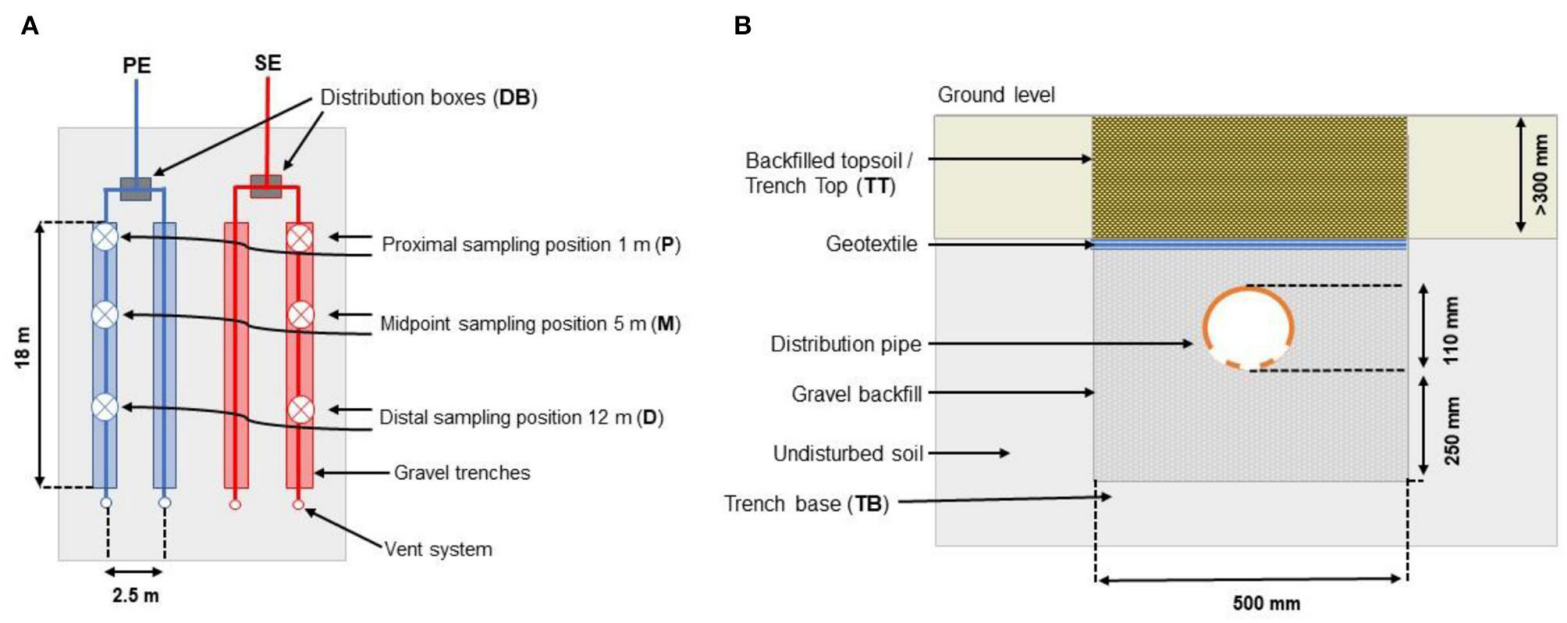
**(A)** Horizontal and **(B)** vertical profile of the STU (Knappe et al., 2020).

This study investigates the spatial distribution of microorganisms in two separate OWTSs located in two owner-occupied homes in Co. Limerick, Ireland. The region is classified as a temperate oceanic climate (or *Cfb* classification within the Köppen climate classification system) (De Carli et al., 2018). Subsoils at both sites are classified as typical Luvisol soils averaging at pH 8 with little observed variance (Fealy, 2009). The microbial biomats have been developed at both research sites, which form at the interface where the effluent percolates into the soil (i.e., the STU) at the base of the gravel percolation trenches. These biomats will be the principle focus of the study. Each percolation trench was 18 m in length and 0.5 m in width, with a gradient of 1:200 filled with 300 mm pea gravel in which a perforated rigid plastic pipe was set (as per EPA, 2021 design guidelines; Figure 1).

The pipes were then covered with 150 mm gravel and a geotextile fabric to prevent backfilled topsoil from washing beneath the gravel layer. Both sites employed a 4,760-L Aswasep two-chambered septic tank (Molloy Precast Products Ltd., Ireland), and four percolation trenches, two of which were fed directly from the septic tank system as the primary effluent (PE), and the other two percolation trenches were fed with the secondary treated effluent (SE) from packaged treatment systems, as shown in Figure 1. At Site A, secondary treatment was achieved using an intermittently dosed coconut husk filter system (Ecoflo Coco Filter, Premier Tech Aqua Ltd., Ireland). At Site B, the packaged secondary treatment system was an RBC (Klargester BioDisc, Kingspan., Ltd, United Kingdom), consisting of an integrated primary settling chamber, a two-stage biozone, and a secondary clarifying chamber. At both sites, the primary and secondary effluents were distributed equally onto their own separate half of the STU. The even distribution of the effluents between these parallel trenches was ensured using calibrated tipping bucket distribution devices (Patel et al., 2008), which were instrumented with reed switches to calculate the daily flows to each STU. The soil and hydrogeological factors from each site (as determined from a parallel research study; Knappe et al., 2020) are summarized in Table 1.

**Table 1:** Soil and hydrological parameters of both sites (Knappe et al., 2020).

**Parameter**	**Site A**	**Site B**
%Sand-Silt-Clay	59–30–11	49–34–17
Bulk density (cm^3^ g^−1^)	1.44	1.2 0
Porosity	0.386	0.448
Groundwater (m)	1.6	>2.5
k_sat_ (cm d^−1^)^a^	30.9	13.9
k_sat__sd	3.5	5.4
Flow mean (Ld^−1^)	269.8	500.1
Flow_sd	329.1	200.8
Construction	September 2015	April 2016
Primary treatment	Septic tank	Septic tank
Secondary treatment	Cocopeat media filter	Rotating biological contactor
Flow regime	Pumped flow	Gravity flow
Number of occupants	5	4

^a^*k_sat_, saturated hydraulic conductivity*.

### Sampling and 16S RRNA Gene Sequencing

At selected locations, the soil was excavated from the surface down into the percolation trenches to the gravel subsoil interface at the base of the trenches. Core samples were then taken with a 25.4-mm stainless steel corer. At each site and for each system, a single sample was taken in each of the three different depths from this infiltrative interface (2.5, 5.0, and 7.5 cm) (Supplementary Figure S2), for each sampling position in the horizontal direction away from the inlet of the STU. A sample was also taken from the surface interface (i.e., 0 cm depth). For DNA extraction of the samples for sequencing, ~3 g of soil was collected from the core and placed into a sterile 2 ml Eppendorf tube and stored at −20°C. Sample handling was performed with a sterile metal spatula, with sterilizing performed between each sample using 70% ethanol. Then, 250 mg of the samples were extracted from the 92 soil samples taken from the field using a DNAeasy power soil kit (Qiagen, NL) (Table 2). DNA concentration was checked using a NanoDrop spectrometer (Nanodrop ND-1000, ThermoScientific, Waltham, MA).

**Table 2:** The sample counts for each on-site wastewater treatment systems for both research sites.

**Location**	**Abbreviation**	**Site A**	**Site B**
Primary effluent	PE	1	1
Secondary effluent	SE	1	0
Primary effluent trench topsoil	PE-TT	6	6
Secondary effluent trench topsoil	SE-TT	6	6
Primary effluent trench subsoil	PE-TB	15	12
Secondary effluent trench subsoil	SE-TB	12	12
Primary effluent distribution box	PE-DB	0	1
Secondary effluent distribution box	SE-DB	0	1
Rotating biodisc contactor	RBC	0	2
Control topsoil	CT	3	3
Control subsoil	CB	2	2

*Total sample counts (n = 92)*.

The microbial community was assessed by next-generation amplicon sequencing of the 16S rRNA in a paired end mode. DNA extracts were sequenced with an Illumina MiSeq platform (NU-OMICS, Northumbria University, United States) using the primer set F515/R806 targeting 294 bp of the V4 region of the bacterial 16S rRNA gene, as described by Kozich et al. (2013).

### Sequence Processing and Analysis

Pair end reads were converted into exact amplicon sequence variant libraries produced using the DADA2 pipeline package with the R program (v1.18.0; Callahan et al., 2016). The trimmed forward and reverse reads were merged with settings −25 M to 230 M. Chimeras were removed with the “removeBimeraDenovo” function in DADA2 under default settings. Taxonomic rank was derived using the “assignTaxonomy” function linked to the Silva database v 138.1. Putative functional groups were identified using the MIDAS database v 4.8.1 (Dueholm et al., 2021) assigned to the Usearch software package v11 (Edgar, 2013).

Alpha diversity (richness and evenness within the samples) was assessed by computing the total number of Operational Taxonomic Units (OTUs), abundance-based coverage estimator (ACE) (Chao and Lee, 1992), and Shannon diversity (Shannon, 1948) for all 92 samples. Principal Coordinate analysis (PCoA) plots were estimated using Bray-Curtis and weighted Unifrac metrics, which were derived from the rarefied OTU table using the ampvis2 v 2.7.11 and phyloseq v 3.6 packages, respectively. Further analysis was performed to determine the categorical variables of statistical significance to determine the variation within the microbial communities. Permutational analysis of variance applying distance matrices (ADONIS) using the vegan package v 2.4.2 (Oksanen et al., 2021) was performed to examine several variables based on 2,000 permutations. All analyses and plots were performed on R version 4.1.1 through the Rstudio IDE (R Core Team, 2014).

### Site Instrumentation

Both research sites were fitted with automated weather stations (Campbell Scientific, United Kingdom) measuring air temperature, relative humidity, atmospheric pressure, net radiation, wind speed and direction, and rainfall. Hydraulic loadings were determined using calibrated tipping buckets (described previously). A network of suction lysimeters (Model 1900, Soilmoisture Equipment Corp., United States) was installed at each site spaced longitudinally along each trench and at three different depths beneath the infiltrative surface down to 50 cm depth in the soil. For sample collection, a suction of 50 kPa was applied using a vacuum-pressure hand pump and samples were collected 24 h later. The effluent and porewater samples extracted from lysimeters were stored on ice for <6 h of transport to be analyzed in the environmental engineering laboratory at Trinity College Dublin. The organic loadings of the PE and SE fed into the STUs were determined as chemical oxygen demand (COD) using dichromate digestion test kits (Merck, Germany) and the total organic carbon (TOC) was determined using a Shimadzu TOC-V analyzer (Shimadzu Scientific Instrument, United States). Nitrogen species were analyzed as nitrate-nitrogen (NO_3_-N), nitrite-N (NO_2_-N), and ammonium–N (NH_4_-N) and phosphorus as ortho-phosphate (PO_4_-P) using a Konelab 20i chemistry analyser (Thermo Scientific, Finland). Assessing the spatial distribution of the volumetric water content (VWC) and long-term changes in water retention within the STUs was achieved by using a network of 80 and 92 soil moisture sensors (EC5, Decagon Devices, United States), which have been installed during the construction of Sites A and B, respectively. Sensors were installed by auguring a 10-cm diameter hole to a desired depth, with sensors positioned into undisturbed subsoil at a required depth below the STU. Control sensors were installed outside of the STU area at the corresponding depths to those below the STU. Calibrations were performed according to the manufacturer's instructions using site-specific subsoils retrieved from test holes excavated prior to the construction of the sites. All sensor data were collected every hour and stored on a CR1000 data logger with two AM15/32 multiplexers (Campbell Scientific, United Kingdom).

### Data Availability Statement

Raw sequencing data were deposited at the National Center for Biotechnology Information (NCBI) under accession number PRJNA794316.

## Results

### Meteorological Conditions

Sites A and B received a mean annual rainfall of 928.6 and 972.4 mm, at mean temperatures of 10.0 and 10.4°C, with low values of −7.2 and −7.4°C and high values of 30.8 and 30.3°C, respectively, over the study period. At the time of sampling in August 2018, the total monthly precipitation was 41.9 and 17.6 mm, at mean temperatures of 15.7 and 15.6°C for Sites A and B, respectively. Detailed trend graphs of total monthly precipitation, monthly air temperatures, and monthly actual evapotranspiration for both sites can be found in the Supplementary Figure S7. Between 19 May and 12 August 2018, Ireland was affected by a drought period resulting in no effective rainfall and severe soil drying (Met Éireann, 2018).

### Effluent Quality and Wastewater Treatment System Performance

The average quality of the effluent from the septic tanks and packaged treatment plants that were feeding the percolation trenches is shown in Table 3. This clearly shows the difference in the effluent quality, particularly between the two packaged treatment systems where the coco-media filter on Site A was only partially nitrifying and removing just 60% of organics, compared to the RBC on Site B which was fully nitrifying and removing >90% of the organics. The mean effluent hydraulic loadings on Site A was 269.8 L/day and on Site B was 500.1 L/day.

**Table 3:** Effluent characteristics for the primary effluent (PE) from the septic tanks and secondary effluent (SE) from the packaged treatment units on Sites A and B over a 3-year period (from Knappe et al., 2020).

**Site**	**Parameter**	**PE**		**SE**		**Mean removal efficiency**
		**Concentration** **(mean ±SD)**	**Load** **(mean ±SD)**	**Concentration** **(mean ±SD)**	**Load** **(mean ±SD)**	
Site A	COD	605.8 ± 240.6 mg L^−1^	163 ± 64.7 g/d	220.5 ± 116.4 mg L^−1^	59.3 ± 31.3 g/d	0.636
	TOC	162.5 ± 82.7 mg L^−1^	43.7 ± 22.2 g/d	63.6 ± 42.3 mg L^−1^	17.1 ± 11.4 g/d	0.609
	TN	167.8 ± 69.0 mg L-1	45.1 ± 18.6 g/d	115.7 ± 44.9 mg L^−1^	31.1 ± 12.1 g/d	0.31
	NH4-N	42.7 ± 54.5 mg L^−1^	11.5 ± 14.7 g/d	13.6 ± 18.2 mg L^−1^	3.7 ± 4.9 g/d	0.681
	NO3-N	1.8 ± 2.5 mg L^−1^	0.5 ± 0.7 g/d	32.9 ± 18.0 mg L^−1^	8.9 ± 4.8 g/d	–
	PO4-P	8.8 ± 6.4 mg L^−1^	2.4 ± 1.7 g/d	8.6 ± 5.8 mg L^−1^	2.3 ± 1.6 g/d	0.023
	Total coliforms	3.45 ×10E6 MPN/100 mL		1.11 ×106 MPN/100 mL		0.49 log10
	E. coli	1.35 ×10E5 MPN/100 mL		8.60 ×104 MPN/100 mL		0.20 log10
Site B	COD	1005.4 ± 192.7 mg L^−1^	502.7 ± 96.4 g/d	51.6 ± 43.5 mg L^−1^	25.8 ± 21.8 g/d	0.949
	TOC	303.6 ± 55.2 mg L^−1^	151.8 ± 27.6 g/d	27.4 ± 16.7 mg L^−1^	13.7 ± 8.4 g/d	0.91
	TN	245.0 ± 23.2 mg L^−1^	122.5 ± 11.6 g/d	23.6 ± 28.9 mg L^−1^	11.8 ± 14.5 g/d	0.904
	NH4-N	68.6 ± 55.7 mg L^−1^	34.3 ± 27.9 g/d	1.9 ± 1.8 mg L^−1^	1 ± 0.9 g/d	0.972
	NO3-N	0.8 ± 0.5 mg L^−1^	0.4 ± 0.3 g/d	8.9 ± 3.8 mg L^−1^	4.5 ± 1.9 g/d	–
	PO4-P	10.3 ± 4.9 mg L^−1^	5.2 ± 2.5 g/d	10.1 ± 5.5 mg L^−1^	5.1 ± 2.8 g/d	0.019
	Total coliforms	7.24 ×106 MPN/100 ml	1.34 ×105 MPN/100 ml	1.73 log10		
	*E. coli*	3.09 ×105 MPN/100 ml	4.09 ×102 MPN/100 ml	2.88 log10		

The two systems at Sites A and B have been in operation for ~35 and 29 months, respectively, when the soil samples from the percolation area were taken. An intensive research study has characterized the performance of the OSWTSs in terms of the three-dimensional attenuation of contaminants as they passed down through the soil, and the effluent quality of the upstream treatment units (septic tanks and secondary treatment units)—as detailed by Knappe et al. (2020). The in-line three-dimensional soil water content sensor network and the chemical quality of soil moisture percolating beneath the STUs provided detailed surveillance of the biomat development within and below the infiltrative soil surface, using water retention as a proxy for the presence of the biomat due to biologging caused by the extracellular polymeric substance matrix present within the biomat. There were significant differences in mean water retention between the STUs receiving the septic tank primary effluent (PE) and packaged treatment system secondary effluent (SE) across all positions. The highest amount of water retention was found at a 5-cm depth closest to the biomat, followed by the upper layer of the infiltrative surface, decreasing with depth.

### Biomat Position

Research performed by Knappe et al. (2020) revealed the spatial variation of water retention using networks of soil moisture sensors. These sensors were employed at the same research sites as this study was effective at determining the position, growth rate, and the effective hydraulic conductivity of the biomats. This was achieved by classifying any region of the STU as an established biomat if it can maintain a mean increase of VWC 0.025 cm^3^ cm^−3^ above the baseline value over a period of 30 days. The biomat position has extended horizontally for 15 m from the inlet for PE-STUs at Sites A and B. Site A exhibited a faster growth rate, with the biomat reaching 15 m in 10 months, and Site B reaching that length in 13 months. Growth was more arrested at the SE-STU with the biomat horizontally extending by 7.5 m and 10 m for Sites A and B after 3 years in operation, respectively. Vertical growth of the biofilm appears to be limited to approximately 5 cm from the infiltrative surface.

### Microbial Community Composition With the STUs

**Figure 2:**
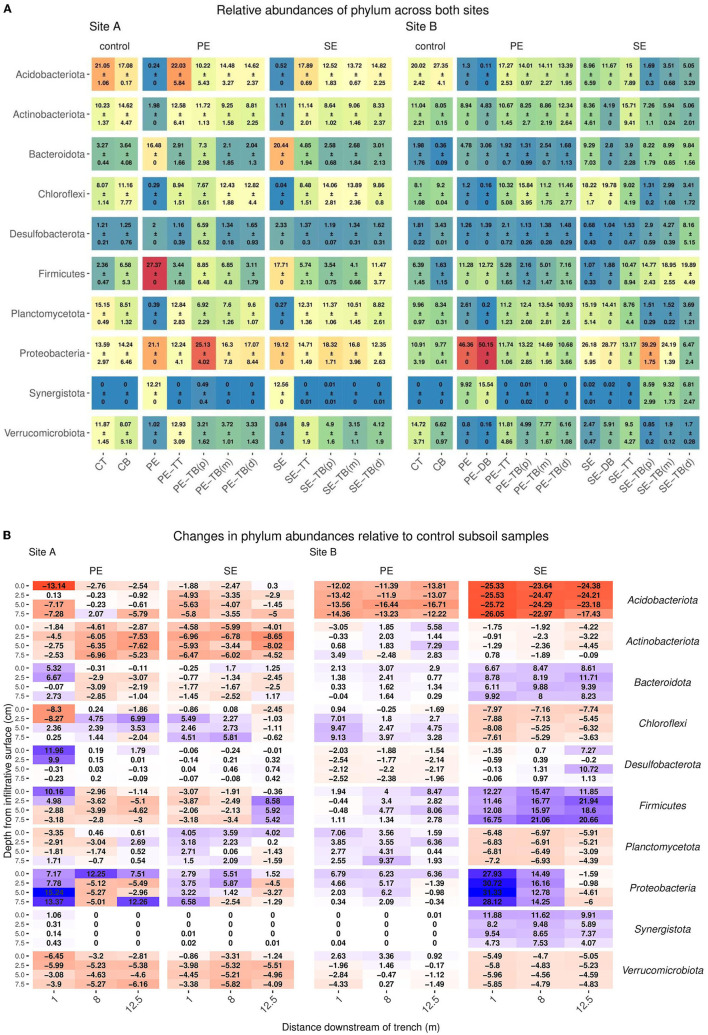
**(A)** Mean ± SD of relative read abundance of phylum level analysis for Sites A and B for both the Primary (PE), secondary (SE), and control samples for each “system”: control; base (CB), top (CT), STU topsoil (TT), and STU subsoil “trench base” (TB). The STU base is further divided into proximal (P) at 1 m, midpoint (m) at 5 m, and distal (d) at 12 m. **(B)** The changes of key phylum relative abundance relative to control subsoil communities, and increases are highlighted in blue and reductions in red.

For the 92 samples sequenced, this resulted in a total of 73,332,182 reads with a read depth per sample ranging from 29,706 (min) to 220,139 (max). Read depth was rarefied to the approximate average value of 35,000 reads per sample. The most abundant phyla in control subsoils for Site A were *Acidobacteriota, Actinobacteriota*, and Proteobacteria (17.08 ± 0.17, 14.62 ± 4.47, and 14.24 ± 6.46, respectively), and for Site B, *Acidobacteriota, Proteobacteria*, and *Chloroflexi* (27.35 ± 4.1, 9.77 ± 0.41, and 11.16 ± 7.77, respectively) as described in Figure 2A (Supplementary Table S3). Changes in abundance of key phyla relative to the control subsoil are described in Figure 2B. Within the STUs there was a pattern of top phyla being composed of *Proteobacteria, Acidobacteria*, and *Chloroflexi* for PE Site A (25.13 ± 4.02, 14.62 ± 2.37, and 12.82 ± 4.4), PE Site B (14.69 ± 1.95, 14.11 ± 2.27, and 15.84 ± 3.95), and SE Site A (18.32 ± 1.71, 14.82 ± 2.25, and 14.06 ± 2.81), respectively. In Site B SE-STU, *Proteobacteria, Firmicutes*, and *Bacteroidetes* were the dominant phyla with abundances of 39.29 ± 1.75, 19.89 ± 4.49, and 9.84 ± 1.56, respectively. *Gammabacteria* consisted of the majority abundances of *Proteobacteria* across all STUs (Supplementary Figure S6).

**Figure 3:**
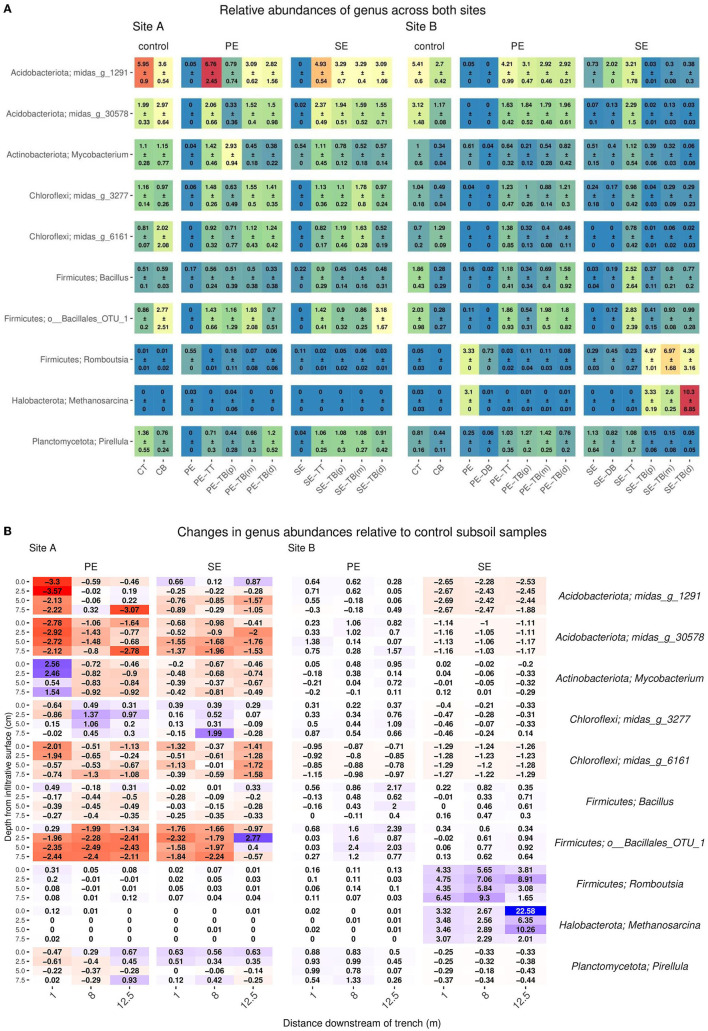
**(A)** Mean ± SD of relative read abundance of genus-level analysis for Sites A and B for both the Primary (PE), secondary (SE), and control samples for each “system”: control; base (CB), top (CT), STU topsoil (TT), and STU subsoil “trench base” (TB). The STU base is further divided into proximal (P) at 1 m, midpoint (m) at 5 m, and distal (d) at 12 m. **(B)** 2D spatial profile of changes of key genus relative abundance with respect to the control subsoil communities; increases are highlighted in blue and reductions in red.

The top 10 most abundant species-level taxa were selected for and compared across compartments within the systems at both sites (Supplementary Table S5). The control subsoil samples at both sites contained high abundances of species belonging to the phylum *Acidobacteriota* (Site A: 3.6 ± 0.54 and Site B: 2.7 ± 0.42), Actinobacteria (Site A: 1.15 ± 0.77 and Site B: 0.51 ± 0.16), and *Chloroflex*i (Site A: 2.02 ± 2.08 and Site B1: 29 ± 0.09) as shown in Figure 3A.

In PE effluent samples, Firmicutes was the most abundant genus, with abundances of 0.91 and 3.33 for Sites A and B, respectively. In the Site B PE samples, *Metanosarcina* sp. and *Thauera* sp. genus were also high with abundances of 3.1 and 5.98, respectively. In the SE fed STUs, *Mycobacterium* sp. (Site A: 0.78 ± 0.12 and Site B: 0.39 ± 0.06) was the most abundant species within both sites, followed by *Pirellula* sp. (1.08 ± 0.3) in Site A and *Bacillus* (0.8 ± 0.21) in Site B. The community profiles for topsoil samples did not vary largely between the samples at both sites and effluent types. At Site A, the phylum *Acidobacteriota* and Actinobacterium abundances were 22.03 ± 5.84 and 12.58 ± 6.41 in the PE-TT, respectively, and abundances in the SE-TT were 17.89 ± 0.69 and 11.14 ± 2.01, respectively. At Site B, the *Acidobacteriota* and Actinobacterium abundances were 17.27 ± 2.53 and 10.67 ± 1.45 in the PE-TT, respectively, and abundances in the SE-TT were 15 ± 7.89 and 15.71 ± 9.41, respectively.

The greatest variance within the microbial community structure was determined from the STU base samples; changes in genus abundances were observed with respect to the distance from the inlet to the trenches and the relative read abundance within the subsoil control sample. At Site A, *Actinobacteriota* species sequences at 1 m along the PE fed trench were higher by an average of 2% with relative read abundance when compared to the control subsoil sample (Figure 3B). For the SE fed STU, *Nitrospira* sp. increased by an average of 1% across all distances sampled (1, 5, and 12 m) along the STU compared to the relative sequence read abundances found within the control subsoil. In the PE-STU, at Site A, there was an average drop of 3% in the read abundance relative species belonging to the Acidobacteriota phylum at 1 m along the trench and an average of 1% at a 12-m sampling point. The parallel SE fed STU showed a 1% reduction across the sampled length of the trench. There was an average drop of 1–2% of Firmicute *Bacillales* and Verrumicrobiota *Chtoniobacterale*s species sequences in both PE and SE-STUs.

In Site B, there was an increase of 2% for Firmicute species sampled at 5–12 m from the head of the trench. The highest increase, in Site B, is observed for the Firmicute species at 2 and 7% for the PE and SE fed STUs, respectively. There were 3-10% increases in *Methanosarcina* sp., 3–4% in *Synergistota* species, and 9% in Proteobacteria *Thauera* sp. within the SE-STU relative to the abundances found within the control subsoil, as shown in Figure 3B. In general, changes in the microbial community composition were more evident at the phylum level with regards to the application of primary, partially, and fully treated effluents, which resulted in a negative response of Acidobacteria and a weak to very strong positive response of Proteobacteria. At a more granular species level, some of the greatest variations in the composition were observed at the SE fed STU, where there were large increases of the relative abundance of *Methanosarcina* sp., *Romboutsia* sp., and *Thauera* sp.

### Biomat Microbial Community Structure in Response to Effluent Dispersal

**Figure 4:**
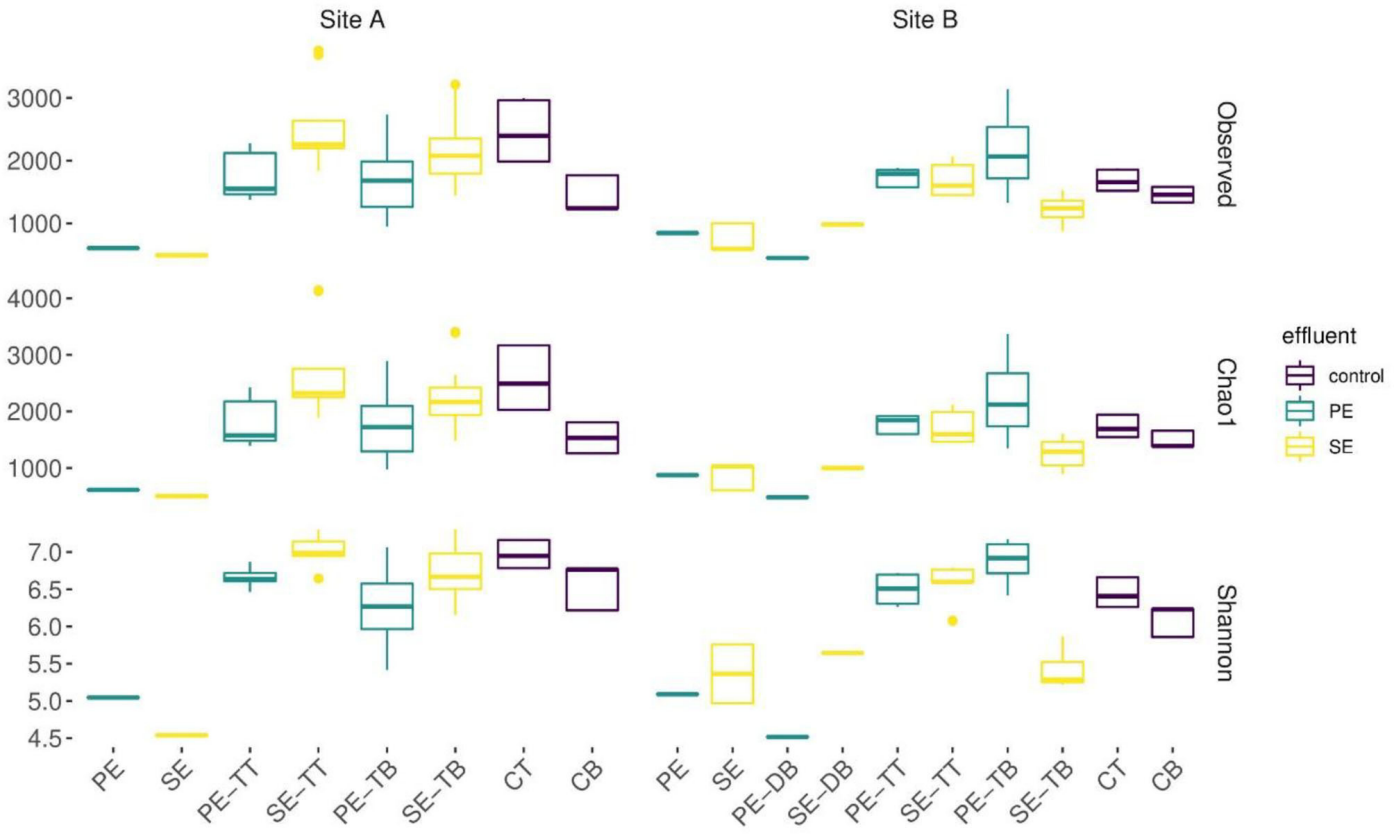
Boxplots displaying rarefied data for the observed OTUs, species richness calculated using an abundance-based coverage estimates (Chao1) and alpha diversity (Shannon). Samples were aggregated on the basis of systems' primary, secondary effluent (PE, SE), distribution box biofilms (DB), STU top (TT), STU base (TB), control top (CT), and base (CB).

By comparing the alpha diversity (Shannon) and species richness (Chao1) between all compartments across both sites, Site A was significantly richer in species (*p* = 0.03, Wilcoxon test), but there was no overall significant difference in diversity (*p* > 0.05, Wilcoxon test). There was no significant difference in the diversity or richness between the control soil samples (Supplementary Figure S3; Supplementary Table S2). Mean diversity values within the PE fed STUs of Site B were significantly greater than those in Site A (*p* = 0.0008; Wilcoxon test). The Site A SE-STU was significantly more rich in species (*p* ≤ 0.0001; Wilcoxon test) and diverse (*p* ≤ 0.0001; Wilcoxon test) than that of Site B. Statistical analysis of alpha diversity and species richness (shown in Figure 4) confirmed that Site A PE fed STU diverged from the control. In Site B, there was a greater significant difference between the SE-STU samples and the other sample groups (Table 4).

**Table 4:** The Wilcoxon test values for comparative intra-site analysis.

	**PE-TB vs. SE-TB**		**PE-TB vs. CB**		**SE-TB vs. CB**	
	**Shannon**	**Chao1**	**Shannon**	**Chao1**	**Shannon**	**Chao1**
Site A	*	**	ns	ns	ns	ns
Site B	****	***	*	ns	*	ns

ns, P > 0.05, *P ≤ 0.05, **P ≤ 0.01, ***P ≤ 0.001 and ****P ≤ 0.0001. Samples were aggregated on the basis of systems primary, secondary effluent (PE, SE), distribution box biofilms (DB), STU top (TT), STU base (TB), control top (CT), and base (CB).

**Figure 5:**
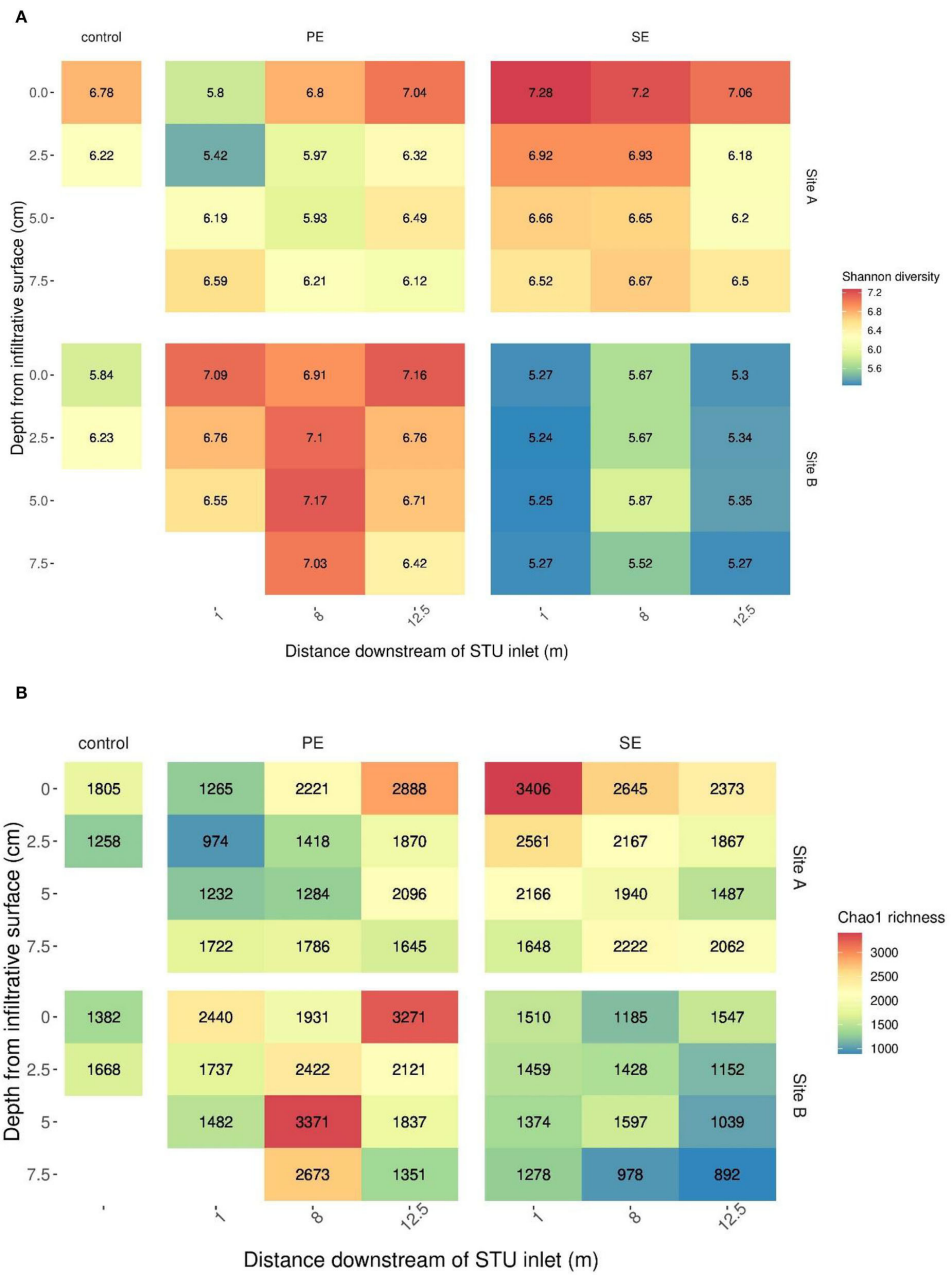
The Shannon diversity **(A)** and Chao1 richness **(B)** across the distance and depth of the sites' primary (PE) and secondary effluent (SE) STUs.

A cross-sectional analysis of horizontal and vertical dimensions of the different STUs across both sites indicated the presence or absence of species richness and diversity “*hotspots*” within both systems (Figure 5).

**Figure 6:**
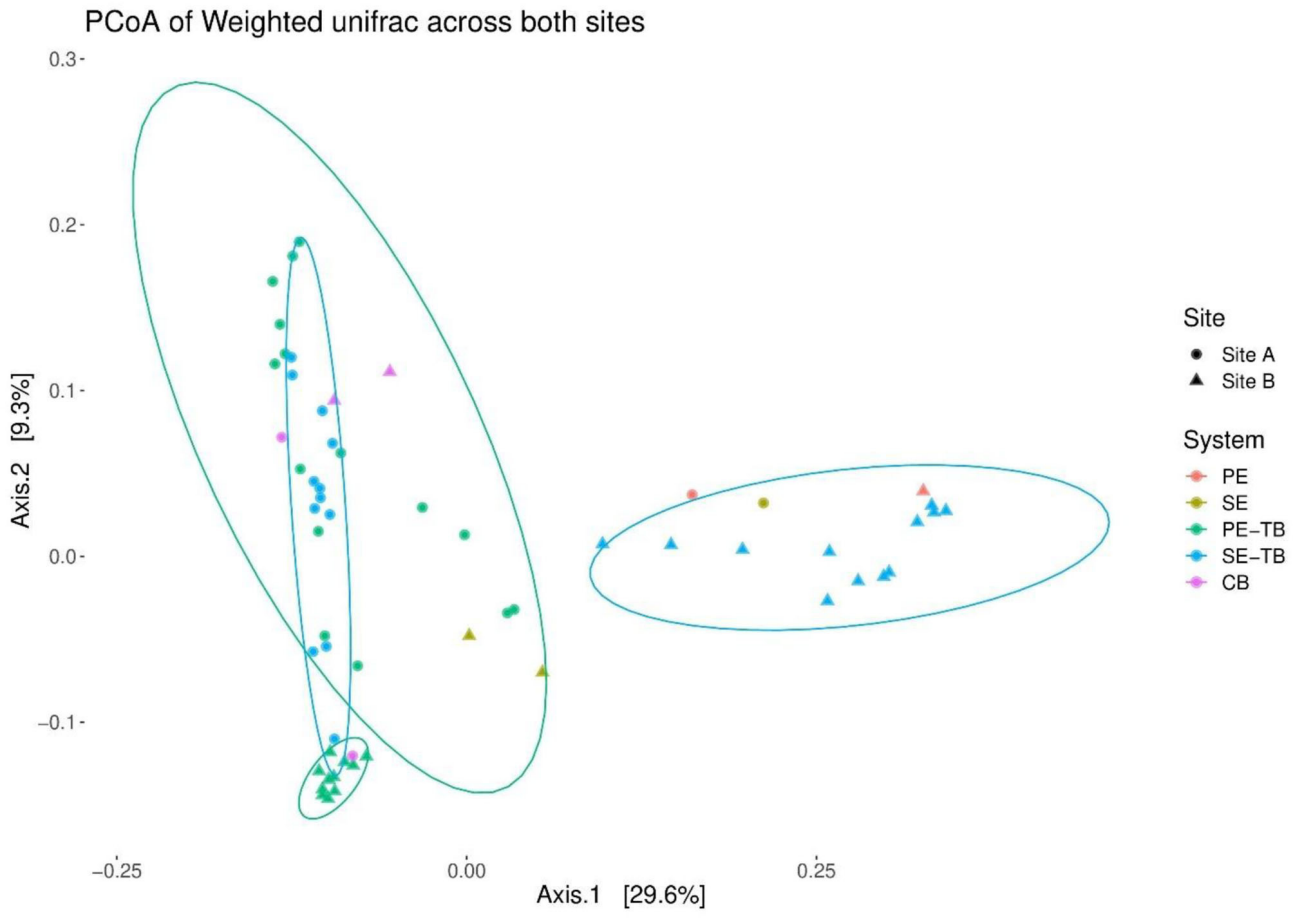
Principal coordinates of beta diversity based on weighted Unifrac distances within STUs at both sites. Each data point represents a sample taken from either PE effluent stream, SE effluent stream, or control soils. Samples are further subdivided based on the position within the system, i.e., at the STU; base, top, and control; base, top, and pure effluent samples.

The PCoA plots were used to investigate the beta diversity using weighted Unifrac, accounting for the relative read abundance within the samples. A large cluster consisting mainly of STU samples is positioned close to the subsoil controls, with the only exception that the PE samples located within 1 m of the STU inlet. This suggests less dissimilarity between the STUs and control subsoil samples for Site A. In contrast, Site B presented much more distinct clusters (Figure 6), with a clustering of shallow subsoil samples with the control soil samples with the PE-STU samples creating a minor cluster. A second separate cluster of the SE-STU samples and the PE effluent sample was present, distinct from the STU, control subsoil, and RBC samples.

Permutational analysis of variance applying distance matrices results indicated that there was no significant difference between the control subsoil communities across sites (Pr = 0.33, R^2^ = 0.49). There were significant differences in the microbial community composition between the topsoil control samples and the subsoil samples (Pr = 0.018; R^2^ = 0.26), and statistically significant differences between the STUs of the same effluent type when compared between the sites (PR = 0.0004; R^2^ = 0.22 and PR = 0.0004; R^2^ = 0.44, for PE and SE, respectively). Table 5 highlights the key factors impacting variance within sites such as effluent and depth, whereas depth did not have any statistically significant effect on the community structure.

**Table 5:** The ADONIS test (ns Pr > 0.05, *Pr ≤ 0.05, **Pr ≤ 0.01, ***Pr ≤ 0.001, and ****Pr ≤ 0.0001) values for comparative intra-site permutational analysis of variance applying distance matrices.

	**PE-TB vs**. **CB**		**SE-TB vs**. **CB**		**PE-TB vs**. **SE-TB**		**PE-TB vs**. **distance (m)**		**SE-TB vs**. **distance (m)**	
	**Pr**	**R^**2**^**	**Pr**	**R^**2**^**	**Pr**	**R^**2**^**	**Pr**	**R^**2**^**	**Pr**	**R^**2**^**
Site A	0.39 ns	0.06	0.23 ns	0.13	0.004**	0.16	0.0004***	0.34	0.001***	0.34
Site B	0.009**	0.32	0.01**	0.38	0.004**	0.49	0.003**	0.36	0.0009***	0.65

*Samples were aggregated on the basis of systems' primary, secondary effluent (PE, SE), STU base (TB), and base (CB)*.

### Target Organisms Screened for Biogeochemical Functionality

**Figure 7:**
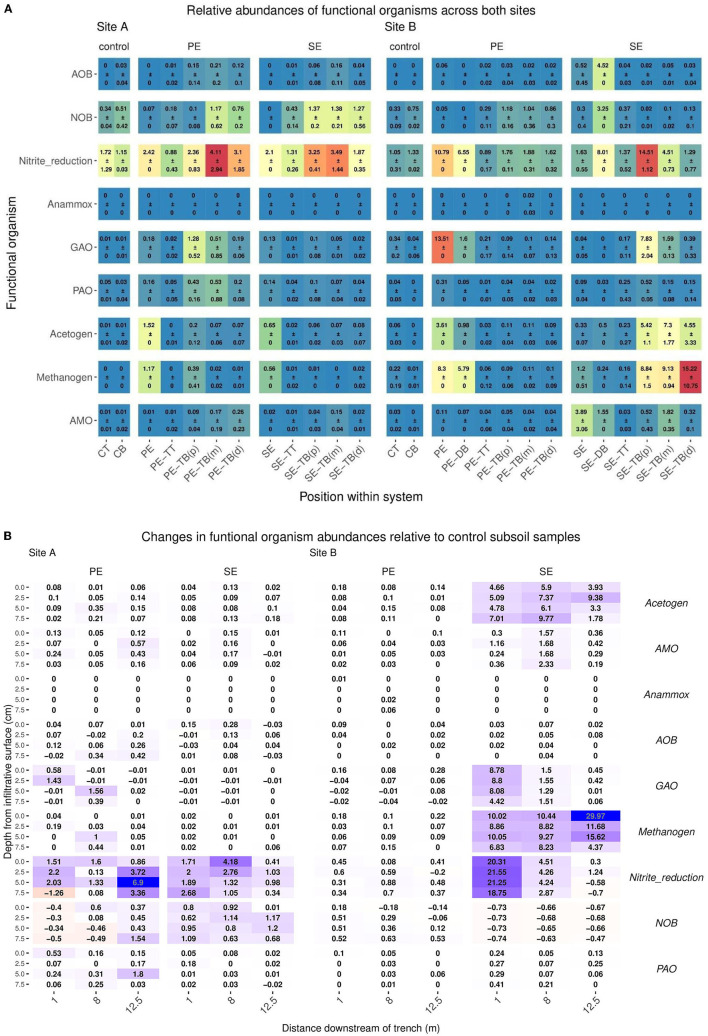
**(A)** Mean ± SD of relative read abundance of Anaerobic Methane Oxidizers (AMO), Denitrifying bacteria, polyphosphate-accumulating organisms (PAO), Nitrite Oxidizing Bacteria (NOB), Methanogens, Glycogen Accumulating Organisms (GAO), Ammonia Oxidizing Bacteria (AOB), AMO, and Acetogen functional groups for Site A for both the Primary (PE), secondary (SE), and control samples for each “system”: control; base (CB), topsoil (CT), STU topsoil (TT), and STU subsoil “trench base” (TB). The STU base is further divided into proximal (P) at 1 m, midpoint (m) at 5 m, and distal (d) at 12 m. **(B)** 2D spatial profile of changes of relative abundances of Anaerobic Methane Oxidizers (AMO), Denitrifying bacteria, Polyphosphate-accumulating organisms (PAO), Nitrite Oxidizing Bacteria (NOB), Methanogens, Glycogen Accumulating Organisms (GAO), Ammonia Oxidizing Bacteria (AOB), AMO, and Acetogen functional groups sequences with respect to the control subsoil communities; increases are highlighted in green and reductions in blue.

Target putative functional groups were classified by means of the MIDAS database, with the only exception being the Anaerobic Methane Oxidizers (AMO) group which was determined from the existing literature. Relative read abundance was measured and compared across sites, STUs, and environmental compartments (topsoil, subsoil), as shown in Figure 7A. Differences in relative abundances for putative functional sequences were compared between the control subsoil samples and STUs for both sites. Key changes in functional groups' relative abundance are summarized in Table 6.

**Table 6:** Key changes (+/–, NC; “No Change”) in relative abundance of functional groups in respect of control subsoil samples.

	**Denitrifying bacteria**	**Nitrite Oxidizing Bacteria (NOB)**	**Polyphosphate-accumulating organisms (PAO)**	**Methanogens**	**Glycogen accumulating organisms (GAO)**	**Ammonia oxidizing bacteria (AOB)**	**AMO**	**Acetogen**	**Anammox**
**Site A**									
PE-STU	(+) 0.08 to 6.9	(+) 0.0.8 to 1.54 (8–12.5 m)	(+) 0.03 to 0.53	(+) 0.01 to 0.44	(+) 0.02 to 1.56 (1–12.5 m)	(+) 0.01 to 0.42 (1–12.5 m)	(+) 0.03 to 0.57	(+) 0.01 to 0.35	NC
	(–) 1.26 (1 m)	(–) 0.3 to 0.5 (1–8 m)			(–) 0.01 (1–12.5 m)	(–) 0.02 (1 m)			
SE-STU	(+) 0.34 to 4.18	(+) 0.01 to 1.17	(+) 0.01 to 0.18 (1–12.5 m)	(+) 0.01 to 0.06	(+) 0.01 (1–12.5 m)	(+) 0.01 to 0.28 (1–12.5 m)	(+) 0.01 to 0.17	(+) 0.02 to 0.18	NC
			(–) 0.02 (12.5 m)		(–) 0.01 (1–12.5 m)	(–) 0.01 to 0.03 (1 m, 8 m)	(–) 0.01 (12.5 m)		
**Site B**									
PE-STU	(+) 0.08 to 0.88	(+) 0.12 to 0.63	(+) 0.01 to 0.1	(+) 0.03 to 0.22	(+) 0.06 to 0.28 (1–12.5 m)	(+) 0.02 to 0.09	(+) 0.01 to 0.11	(+) 0.01 to 0.18	(+) 0.01 to 0.06
	(–) 0.02 (12.5 m)	(8 m, 12.5 m) (–) 0.06 to 0.18 (8 m–12.5 m)			(–) 0.01 to 0.04 (1–12.5 m)				
SE-STU	(+) 0.3 to 21.55 (1–12.5 m)	(–) 0.47 to 0.73	(+) 0.06 to 0.41	(+) 4.37 to 29.97	(+) 0.01 to 8.78	(+) 0.02 to 0.08	(+) 0.19 to 2.33	(+) 1.78 to 9.77	NC
	(–) 0.58 to 0.77 (12.5 m)								

*Samples were aggregated on the bases of systems primary, secondary effluent (PE, SE), and Soil Treatment Unit (STU)*.

The STUs dosed with Primary effluents, Denitrifying bacteria, Polyphosphate-Accumulating Organisms (PAO), Nitrite Oxidizing Bacteria (NOB), Methanogens, Glycogen Accumulating Organisms, Ammonia Oxidizing Bacteria (AOB), AMO and Acetogen functional groups showed higher relative abundances when compared to the control subsoil sample (Table 6). There were decreases in the abundances for Denitrifiers, NOB, GAO, for site A and B, AOB functional groups also decreased in site B (Table 6). The Site B PE fed STU samples also showed the presence of Anammox species sequences at relative abundances of 0.01, 0.02, and 0.06 located in the sampling positions of 1 m distance at 0 cm depth of the infiltrative surface, 5 m distance at 5 cm depth of the infiltrative surface, and 5 m distance at 7.5 cm depth of the infiltrative surface (Table 6; Figure 7), respectively.

For the SE fed STU samples, all functional groups (Denitrifying bacteria, PAO, Methanogen, GAO, AOB, AMO, and Acetogen) showed an increase in relative read abundance with respect to the control subsoil sample. The only notable reductions were the Denitrifiers and NOB functional groups in Site B, with reductions of 0.58–77 and 0.47–0.73 respectively, lower than the abundances found within the subsoil control samples (Table 6). In the SE-STU at Site B, there were large increases in the relative abundance of Denitrifiers, Methanogens, and Acetogens (Table 6). For Denitrifiers and GAOs, higher increases were located around the sampling points proximal to the inlet of the SE fed STU at 1 m position (Figure 7B). Methanogen increases in relative abundances were at a maximum at the rear of the STU at 12.5 m of the inlets whilst the maximum Acetogen relative abundances were observed at the midpoint of the STU at 5 m (Figure 7B).

## Discussion

The main aim of this study was to determine the influence of different levels of treated domestic wastewater on the microbial community structure of soil treatment units (STUs). Two research sites were selected due to their proximity of each other, being in the same climate region and having the same the soil type. Both sites are classified as typical Luvisol soils, averaging at pH 8 with Little known variance (although not measured in this study) and both sites also employ similar land management practices (rural, unproductive domestic households) (Fierer and Jackson, 2006; Fealy, 2009; Karimi et al., 2020). The lack of a significant difference in the species richness, alpha diversity, and the overall community composition of the subsoil control samples between both sites proved the proximity to be effective to allow a direct comparison of the STU systems. Pre-treatment had a significant effect on the alpha diversity and the richness of species between STUs across sites. Between SE-STUs Site A was significantly richer and more diverse in species even though its biomat was 2.5 m shorter than Site B. This shorter biomat is likely because of the greater pore size and the higher k_sat_ values measured at Site A. Variations in richness and species diversity in PE-STUs were less significant and can be attributed to soil pore size and average loading rates which has also been known to impact the growth rate of the biofilm (Bastida et al., 2017; Dang et al., 2019; Knappe et al., 2020).

The 2D spatial analysis located areas of elevated alpha diversity and species richness, which offer important insights into microbial hotspots within the STU. Areas of low richness within the trench may suggest areas of high activity as seen in previous studies of microbial activity within the rhizosphere which are characterized by low species richness (Reinhold-Hurek et al., 2015). A recent study on structural equation modeling (Bastida et al., 2021) has indicated that soil C content plays a role in regulating soil microbial richness by a positive association with microbial biomass (Geyer and Barrett, 2019). High levels of richness and alpha diversity relative to control subsoils were found in locations across the STUs with the exception of Site B SE-STU. Areas with high levels of species richness may be in response to the high nutrient conditions which suggest locations in which organic carbon may have been incorporated into the EPS for the development of the biomat, with its high energy requirements for its production but results in less biomass (Wu et al., 2019). The low richness suggests the end of SE-STUs where the effluent is likely to incur less resistance due to the absence of bioclogging which may be areas of high activity (Knappe et al., 2020).

The sampling campaign allowed us to assess the spatial effect of pre-treatment on alpha diversity and the taxa in the STU. The productivity diversity relationship hypothesizes that once diversity has increased beyond a certain threshold due to resource availability the diversity outcome becomes negative (Geyer and Barrett, 2019). At the proximal position of the PE-STU of Site A there is a reduced diversity relative to the rest of the STU, and an increase of abundance of copiotrophic phyla specifically proteobacteria indicating that this portion of the STU may have tipped a threshold. In Site B PE-STU, the diversity appears to be higher than in Site A, and the increases in copiotrophic organisms were less pronounced, which suggests that the younger biomat with a lower growth rate has not yet tipped into a homogenous community (Knappe et al., 2020). The spatial profile of the taxa proteobacteria, specifically the class gammaproteobacteria, accurately mirrors the position of the biomats across all sites due to its positive response to organic loading in subsoils, however, spatial accuracy is lost further down the taxonomical levels (Dang et al., 2019; Wu et al., 2019). Interestingly, in the SE-STU at Site B, the large increase of gammaproteobacteria taxa relative to the control subsoil at the proximal sampling point (1 m) indicates that the degree of pre-treatment may enhance the competition. The selection for copiotrophic bacteria in the SE-STU at Site B is likely due to the steady flow of limited nutrients and organic inputs and a lack of pressure for resource complementarity (Naeem, 2009).

Permutational multivariate analysis determined that the level of pre-treatment of the effluent had a significant impact on the community structure of the STUs. The clear divergence of the SE-STU from Site B from the remaining systems illustrates the variance caused by the level of pre-treatment (Guo et al., 2018; Knappe et al., 2020). Permutational multivariate analysis also confirmed that spatial factors such as the horizontal distance accounted for a great degree of the variance in community structures, with the SE-STUs being particularly affected. The large variance within the communities of the SE-STUs highlights the presence of “infiltrative dead zones” located at the distal location of the underused trench, resulting in a heterogeneous community composition within the distal portion of the STU (Knappe et al., 2020). The effect of subsoil depth was insignificant on the community structure, which is contrary to several biogeographical surveys of natural subsoils (Eilers et al., 2012; Uksa et al., 2015; He et al., 2017). The lack of any significant effect of the vertical distance from the infiltrative surface may be due to the depth of the soil cores, which at a depth of 7.5 cm may have been insufficient to assess the true diversity of the vertical profile. All depths sampled only encompassed areas of the infiltrative surface impacted by the biomat. Future studies should incorporate deeper cored samples to confirm the true effect of depth on the STU.

This study identified ammonium oxidation (annamox) bacteria *Candidatus brocadia anammoxidans* in the PE fed STU of Site B. *Candidatus anammoximicrobium* was also detected in the effluent of the RBC. The presence of annamox in the PE-STU corresponds to site descriptions reported by Knappe et al. (2020) who confirmed ponded anaerobic conditions within the PE-STU at Site B. Annamox reactions have been of interest as a low energy alternative for nitrogen removal within wastewater treatment plants but are also known to be naturally active within soils and wetlands (Kartal et al., 2010; Bagnoud et al., 2020). The presence of annamox may be due to high concentrations of organic carbon in the STU resulting in concurrent denitrification with heterotrophic Denitrifiers (Chamchoi et al., 2008). These results match previous studies which suggested that anammox was active within low flow sites and at 5–7.5 cm depths within the infiltrative surface (Cooper et al., 2016; Humphrey Jr et al., 2019). Increases in functional richness may help locate metabolic activities such as the presence of denitrifiers at the proximal position to the inlet at the SE-STU (Louca et al., 2018). However, an increase in functional richness which resulted in effective attenuation was not evident in this study. Pre-treatment often results in high nitrate and low organic carbon effluents which increase the relative read abundance of denitrifiers, but a stunted biomat with low hydraulic retention time results in a significantly reduced attenuation of TN (Gill et al., 2009; Knappe et al., 2020; Dubber et al., 2021). Our study has shown that the PE-STUs have less pronounced increases in denitrifiers compared to the SE-STUs, but STUs receiving primary effluents have been found to be capable of removing six times the amount of total nitrogen (Gill et al., 2009). This suggests that the increases in functional richness within the STU are secondary to bioclogging, as metabolic rates could be limited by hydraulic conductivity.

## Conclusion

- This study presents the first direct microbial comparative analysis between STUs for on-site wastewater treatment systems receiving domestic effluents with different levels of pre-treatment. This analysis has been conducted in STUs which have already been successfully characterized for soil clogging, under the same environmental, hydrological, and subsoil conditions.
- The microbial community richness and diversity within the STU system were significantly affected by the level of pre-treatment of wastewater. This outcome appears to follow the productivity diversity relationship theory that, over time, the addition of organics to these systems results in low diversity communities. The effect is not linear and the addition of a steady flow of lower concentrations of organics and nutrients may be more selective for copiotrophic bacteria than for the raw effluent.
- The STU receiving the fully pre-treated effluent contained the highest relative abundance of Denitrifying bacteria, however the increases in functional richness may not indicate the attenuating capacity of the system. Attenuation appears to be linked to the extent of the coverage of the STU by the biomat. This suggests that the increases in functional richness within the STU are secondary to bioclogging, as metabolic rates could be limited by hydraulic conductivity.
- The community structure or beta diversity of the STUs was significantly impacted by the level of pre-treatment and the horizontal distance downstream of the inlet. Depth did not significantly impact community structure in any of the STUs.
- This study has effectively profiled two developed and stunted biomats within the STUS of two separate research sites. More temporal data are required to assess the development of these communities in the field. In doing so, it will be possible to establish the critical points of transition for the STUs, profiling communities at different stages of the growth of the biomat which will provide researchers with in-depth understanding of the biological clogging process and how to manage it.

## Data Availability Statement

The datasets presented in this study can be found in online repositories. The names of the repository/repositories and accession number(s) can be found below: NCBI BioProject—PRJNA794316.

## Author Contributions

JK and LG devised the study design. Fieldwork was performed by JK, CS, CB, and LG. Lab work was performed by AC and JK and coordinated by TC and LG. Data analysis was performed by AC under the guidance of JK, CB, MA, and LG. The first draft was written by AC. All authors contributed to the revision of the manuscript and approved the submission of the manuscript.

## Funding

The funding for the study was provided by the Irish Research Council (IRC) as a Starting Laureate Award (Grant Number IRCLA/2017/246). The funding source had no role in the design and execution of this study, analysis, and interpretation of the data, or decision to submit results.

## Conflict of Interest

The authors declare that the research was conducted in the absence of any commercial or financial relationships that could be construed as a potential conflict of interest.

## Publisher's Note

All claims expressed in this article are solely those of the authors and do not necessarily represent those of their affiliated organizations, or those of the publisher, the editors and the reviewers. Any product that may be evaluated in this article, or claim that may be made by its manufacturer, is not guaranteed or endorsed by the publisher.

## References

[B1] Ali M. Wang Z. Salam K. W. Hari A. R. Pronk M. van Loosdrecht M. C.. (2019). Importance of species sorting and immigration on the bacterial assembly of different-sized aggregates in a full-scale aerobic granular sludge plant. Environ. Sci. Technol. 53, 8291–8301. 10.1021/acs.est.8b07303 31194515

[B2] Amador J. A. Loomis G. W. (2019). Soil-Based Wastewater Treatment. New York: Wiley. 10.2134/sbwtreatment

[B3] Bagnoud A. Guye-Humbert S. Schloter-Hai B. Schloter M. Zopfi J. (2020). Environmental factors determining distribution and activity of anammox bacteria in minerotrophic fen soils. FEMS Microbiol. Ecol. 96, fiz191. 10.1093/femsec/fiz191 31782780

[B4] Bastida F. Eldridge D. J. García C. Kenny Png G. Bardgett R. D. Delgado-Baquerizo M. (2021). Soil microbial diversity-biomass relationships are driven by soil carbon content across global biomes. ISME J. 15, pp.2081–2091. 10.1038/s41396-021-00906-0 33564112 PMC8245509

[B5] Bastida F. Torres I. F. Romero-Trigueros C. Baldrian P. Větrovsk,ý T. Bayona J. M.. (2017). Combined effects of reduced irrigation and water quality on the soil microbial community of a citrus orchard under semi-arid conditions. Soil Biol. Biochem. 104, 226–237. 10.1016/j.soilbio.2016.10.024

[B6] Beal C. D. Gardner E. A. Menzies N. W. (2005). Process, performance, and pollution potential— a review of septic tank soil absorption systems. Aust. J. Soil Res. 43, 781–802. 10.1071/SR05018

[B7] Bouma J. (1975). Unsaturated flow during soil treatment of septic tank effluent. J. Environ. Eng. Div. 101 967–983. 10.1061/JEEGAV.0000438

[B8] Callahan B. J. McMurdie P. J. Rosen M. J. Han A. W. Johnson A. J. A. Holmes S. P. (2016). DADA2: High-resolution sample inference from Illumina amplicon data. Nat. Methods 13, 581–583. 10.1038/nmeth.3869 27214047 PMC4927377

[B9] Chamchoi N. Nitisoravut S. Schmidt J. E. (2008). Inactivation of ANAMMOX communities under concurrent operation of anaerobic ammonium oxidation (ANAMMOX) and denitrification. Bioresour. Technol. 99, 3331–3336. 10.1016/j.biortech.2007.08.029 17911013

[B10] Chao A. Lee S. M. (1992). Estimating the number of classes via sample coverage. J. Am. Stat. Assoc. 87, 210–217. 10.2307/2290471

[B11] Chen Y. Lan S. Wang L. Dong S. Zhou H. Tan Z.. (2017). A review: driving factors and regulation strategies of microbial community structure and dynamics in wastewater treatment systems. Chemosphere 174, 173–182. 10.1016/j.chemosphere.2017.01.129 28161518

[B12] Cooper R. J. Fitt P. Hiscock K. M. Lovett A. A. Gumm L. Dugdale S. J.. (2016). Assessing the effectiveness of a three-stage on-farm biobed in treating pesticide contaminated wastewater. J. Environ. Manage. 181, 874–882. 10.1016/j.jenvman.2016.06.047 27397841

[B13] Curtis T. P. Sloan W. T. (2005). Exploring microbial diversity—a vast below. Science. 309, 1331–1333. 10.1126/science.1118176 16123290

[B14] Daims H. Taylor M. W. Wagner M. (2006). Wastewater treatment: a model system for microbial ecology. Trends Biotechnol. 24, 483–489. 10.1016/j.tibtech.2006.09.002 16971007

[B15] Dang Q. Tan W. Zhao X. Li D. Li Y. Yang T.. (2019). Linking the response of soil microbial community structure in soils to long-term wastewater irrigation and soil depth. Sci. Total Environ. 688, 26–36. 10.1016/j.scitotenv.2019.06.138 31233911

[B16] De Carli M. Bernardi A. Cultrera M. Dalla Santa G. Di Bella A. Emmi G.. (2018). A database for climatic conditions around Europe for promoting GSHP solutions. Geosciences 8, 71. 10.3390/geosciences8020071

[B17] Dottorini G. Michaelsen T. Y. Kucheryavskiy S. Andersen K. S. Kristensen J. M. Peces M.. (2021). Mass-immigration determines the assembly of activated sludge microbial communities. Proc. Nat. Acad. Sci. USA 118, 589118. 10.1073/pnas.2021589118 34187887 PMC8271747

[B18] Dubber D. Knappe J. Gill L. W. (2021). Characterisation of organic matter and its transformation processes in on-site wastewater effluent percolating through soil using fluorescence spectroscopic methods and parallel factor analysis (PARAFAC). Water 13, 2627. 10.3390/w13192627

[B19] Dueholm M. S. Nierychlo M. Andersen K. S. Rudkjøbing V. Knutsson S. the Mi,DA. S.. (2021). MiDAS 4: a global catalogue of full-length 16S rRNA gene sequences and taxonomy for studies of bacterial communities in wastewater treatment plants. bioRxiv 2021.07.06.451231. 10.1101/2021.07.06.45123135393411

[B20] Edgar R. (2013). UPARSE: highly accurate OTU sequences from microbial amplicon reads. Nat. Methods 10, 996–998. 10.1038/nmeth.2604 23955772

[B21] Eilers K. G. Debenport S. Anderson S. Fierer N. (2012). Digging deeper to find unique microbial communities: the strong effect of depth on the structure of bacterial and archaeal communities in soil. Soil Biol. Biochem. 50, 58–65. 10.1016/j.soilbio.2012.03.011

[B22] EPA (2021). Code of Practice for Domestic Waste Water Treatment Systems (Population Equivalent ≤ 10). Ireland: Environmental Protection Agency.

[B23] Fealy R. (2009). Teagasc-EPA Soils and Subsoils Mapping Project: Final Report V. 1. Teagasc, Environmental Protection Agency.

[B24] Fierer N. Jackson R. B. (2006). The diversity and biogeography of soil bacterial communities. Proc. Natl. Acad. Sci. USA. 103, 626–631. 10.1073/pnas.0507535103 16407148 PMC1334650

[B25] Frigon D. Wells G. (2019). Microbial immigration in wastewater treatment systems: analytical considerations and process implications. Curr. Opin. Biotechnol. 57, 151–159. 10.1016/j.copbio.2019.02.021 31030172

[B26] Geyer K.M. Barrett J.E. (2019). Unimodal productivity-diversity relationships among bacterial communities in a simple polar soil ecosystem. Environ. Microbiol., 21, 2523–2532. 10.1111/1462-2920.14639 31020762

[B27] Gill L. W. (2011). The development of a code of practice for single house on-site wastewater treatment in Ireland. Water Sci. Technol. 64, 677–683. 10.2166/wst.2011.685 22097047

[B28] Gill L. W. O'Luanaigh N. Johnston P. M. Misstear B. D. R. Ó'Súlleabháin C. (2009). Nutrient loading on subsoils from on-site wastewater effluent, comparing septic tank and secondary treatment systems. Water Res. 43, 2739–2749. 10.1016/j.watres.2009.03.024 19349058

[B29] Gill L. W. O'Súlleabháin C. Misstear B. D. R. Johnston P. J. (2007). The treatment performance of different subsoils in Ireland receiving on-site wastewater effluent. J. Environ. Qual. 36, 1843–1855. 10.2134/jeq2007.0064 17965387

[B30] Guo Y. S. Furrer J. M. Kadilak A. L. Hinestroza H. F. Gage D. J. Cho Y. K.. (2018). Bacterial extracellular polymeric substances amplify water content variability at the pore scale. Front. Environ. Sci. 93. 10.3389/fenvs.2018.00093

[B31] He S. Guo L. Niu M. Miao F. Jiao S. Hu T.. (2017). Ecological diversity and co-occurrence patterns of bacterial community through soil profile in response to long-term switchgrass cultivation. Sci. Rep. 7, 3608. 10.1038/s41598-017-03778-7 28620188 PMC5472595

[B32] Hu M. Wang X. Wen X. Xia Y. (2012). Microbial community structures in different wastewater treatment plants as revealed by 454-pyrosequencing analysis. Bioresour. Technol. 117, 72–79. 10.1016/j.biortech.2012.04.061 22609716

[B33] Humphrey Jr C. P. Iverson G. Underwood W. J. Cary S. S. Skibiel C. O'Driscoll M. (2019). Nitrogen treatment in soil beneath high-flow and low-flow onsite wastewater systems. J. Sustain. Water Built Environ. 5, 04019006. 10.1061/JSWBAY.0000888

[B34] Karimi B. Villerd J. Dequiedt S. Terrat S. Chemidlin-Prévost Bouré N. Djemiel C.. (2020). Biogeography of soil microbial habitats across France. Glob. Ecol. Biogeogr. 29, 1399–1411. 10.1111/geb.13118

[B35] Kartal B. Kuenen J. V. Van Loosdrecht M. C. M. (2010). Sewage treatment with anammox. Science 328, 702–703. 10.1126/science.1185941 20448175

[B36] Knappe J. Somlai C. Fowler A. C. Gill L. W. (2020). The influence of pre-treatment on biomat development in soil treatment units. J. Contam. Hydrol. 232, 103654. 10.1016/j.jconhyd.2020.103654 32504864

[B37] Knisz J. Shetty P. Wirth R. Maróti G. Karches T. Dalkó I.. (2021). Genome-level insights into the operation of an on-site biological wastewater treatment unit reveal the importance of storage time. Sci. Total Environ. 766, 144425. 10.1016/j.scitotenv.2020.144425 33418265

[B38] Kozich J. J. Westcott S. L. Baxter N. T. Highlander S. K. Schloss P. D. (2013). Development of a dual-index sequencing strategy and curation pipeline for analyzing amplicon sequence data on the MiSeq Illumina sequencing platform. Appl. Environ. Microbiol. 79, 5112–5120. 10.1128/AEM.01043-13 23793624 PMC3753973

[B39] Lee S. H. Kang H. J. Park H. D. (2015). Influence of influent wastewater communities on temporal variation of activated sludge communities. Water Res. 73, 132–144. 10.1016/j.watres.2015.01.014 25655320

[B40] Louca S. Polz M. F. Mazel F. Albright M. B. N. Huber J. A. O'Connor M. I.. (2018). Function and functional redundancy in microbial systems. Nat. Ecol. Evol. 2, 936–943. 10.1038/s41559-018-0519-1 29662222

[B41] Matar G. K. Ali M. Bagchi S. Nunes S. Liu W.-T. Saikaly P. E. (2021). Relative importance of stochastic assembly process of membrane biofilm increased as biofilm aged. Front. Microbiol. 12, 708531. 10.3389/fmicb.2021.708531 34566913 PMC8461090

[B42] Met Éireann (2018). A Summer of Heat Waves and Droughts. Dublin: Irish Meteorological Service.

[B43] Naeem S. (2009). Biodiversity, Ecosystem Functioning, and Human Wellbeing an Ecological and Economic Perspective. Oxford, UK: Oxford University Press.

[B44] Oksanen J. A. R. I. Blanchet F. G. Friendly M. Kindt R. Legendre P. McGlinn. (2021). Vegan: Community Ecology Package. R package version 2.5–7. 2020.

[B45] O'Luanaigh N. D. Gill L. W. Misstear B. D. R. Johnston P. M. (2012). The attenuation of microorganisms in on-site wastewater effluent discharged into highly permeable subsoils. J. Contam. Hydrol. 142, 126–139. 10.1016/j.jconhyd.2011.12.003 22300802

[B46] Patel T. O'Luanaigh N. Gill L. W. (2008). A comparison of gravity distribution devices used in on-site domestic wastewater treatment systems. Water Air Soil Pollut. 191, 55–69. 10.1007/s11270-007-9606-7 18701801

[B47] R Core Team (2014). R: A Language and Environment for Statistical Computing. Vienna: R Foundation for Statistical Computing.

[B48] Reinhold-Hurek B. Bünger W. Burbano C. S. Sabale M. Hurek T. (2015). Roots shaping their microbiome: global hotspots for microbial activity. Annu. Rev. Phytopathol. 53, 403–424. 10.1146/annurev-phyto-082712-102342 26243728

[B49] Ross B. N. Wigginton S. K. Cox A. H. Loomis G. W. Amador J. A. (2020). Influence of season, occupancy pattern, and technology on structure and composition of nitrifying and denitrifying bacterial communities in advanced nitrogen-removal onsite wastewater treatment systems. Water 12, 2413. 10.3390/w12092413

[B50] Sanz J. L. Köchling T. (2007). Molecular biology techniques used in wastewater treatment: an overview. Process Biochem. 42, 119–133. 10.1016/j.procbio.2006.10.003

[B51] Shannon C. E. (1948). A mathematical theory of communication. Bell Syst Tech J. 27:379–423.

[B52] Siegrist R. L. (2017). “Treatment using subsurface soil infiltration,” in Decentralized Water Reclamation Engineering (Berlin: Springer International Publishing AG), 547–639. 10.1007/978-3-319-40472-1_11

[B53] Siegrist R. L. Boyle W. C. (1987). Wastewater-induced soil clogging development. J. Environ. Eng. 113, 550–566. 10.1061/(ASCE)0733-9372(1987)113:3(550)

[B54] Siezen R. J. Galardini M. (2008). Genomics of biological wastewater treatment. Microb. Biotechnol. 1, 333. 10.1111/j.1751-7915.2008.00059.x 21261852 PMC3815239

[B55] Tomaras J. Sahl J. W. Siegrist R. L. Spear J. R. (2009). Microbial diversity of septic tank effluent and a soil biomat. Appl. Environ. Microbiol. 75, 3348–3351. 10.1128/AEM.00560-08 19304840 PMC2681617

[B56] Truu M. Juhanson J. Truu J. (2009). Microbial biomass, activity and community composition in constructed wetlands. Sci. Total Environ. 407, 3958–3971. 10.1016/j.scitotenv.2008.11.036 19157517

[B57] Uksa M. Schloter M. Kautz T. Athmann M. Köpke U. Fischer D. (2015). Spatial variability of hydrolytic and oxidative potential enzyme activities in different subsoil compartments. Biol. Fert. Soils 51, 517–521. 10.1007/s00374-015-0992-5

[B58] van Cuyk S. Siegrist R. L. (2007). Virus removal within a soil infiltration zone as affected by effluent composition, application rate, and soil type. Water Res. 41, 699–709. 10.1016/j.watres.2006.07.021 16963100

[B59] Wagner M. Nielsen P. H. Loy A. Nielsen J. L. Daims H. (2006). Linking microbial community structure with function: fluorescence in situ hybridization-microautoradiography and isotope arrays. Curr. Opin. Biotechnol. 17, 83–91. 10.1016/j.copbio.2005.12.006 16377170

[B60] Wigginton S. K. Brannon E. Q. Kearns P. J. Lancellotti B. V. Cox A. Moseman-Valtierra S.. (2020). Nitrifying and denitrifying microbial communities in centralized and decentralized biological nitrogen removing wastewater treatment systems. Water 12, 1688. 10.3390/w12061688

[B61] Wu Y. Cai P. Jing X. Niu X. Ji D. Ashry N. M.. (2019). Soil biofilm formation enhances microbial community diversity and metabolic activity. Environ. Int. 132, p.105116. 10.1016/j.envint.2019.105116 31479959

[B62] Zhang Q. Lv X. Wei C. Lu W. Wang J. Zhou Z. Chang G. Gao T. Zhang H. (2020). Microbial community structure diversity in the dewatered sludge from 4 different waste water treatment plants used for CSRB in colder season. E3S Web Conf. 194, 3–6. 10.1051/e3sconf/202019404063

