# Supplementary material for: Spatial Variation of the Microbial Community Structure of On-Site Soil Treatment Units in a Temperate Climate, and the Role of Pre-treatment of Domestic Effluent in the Development of the Biomat Community
Source: Front Microbiol. 2022 Jun 24;13:915856. doi: 10.3389/fmicb.2022.915856 (PMC9263727; doi:10.3389/fmicb.2022.915856)
Supplement: Supplementary file 1 [file Data_Sheet_1.docx]

# Supplementary Information

**Figures**

**S1** Histogram of sequence reads across all samples

**S2** Accessing STU biomat, gravel matrix and percolation pipe visible (right) and sampling using corer

S3 Boxplots displaying rarefied data for observed OTUs, species richness calculated using an abundance-based coverage estimates (Chao1) and alpha diversity (Shannon). Samples were aggregated on the bases of sites

**S4** Means in concentration of ammonium, nitrate, ortho-phosphate, total nitrogen and total organic carbon within the porewater samples at site A, throughout the year of sampling.

**S5** Means in concentration of ammonium, nitrate, ortho-phosphate, total nitrogen and total organic carbon within the porewater samples at site B, throughout the year of sampling.

**S6** changes in relative abundance of Gammaproteobacteria and Alphaproteobacteria relative to the control subsoils.

**Tables**

**S1** Summary of sequence read data

**S2** Table S2 displays the Wilcoxon test values for inter-site analysis Samples were aggregated on the bases of systems primary, secondary effluent (PE, SE)

**S3** Mean relative abundance of key phyla within site A and site B,

**S4** Relative abundance of key genus within site A and site B

Table S1. Summary of sequence read data

| Min | Median | Mean | Max | 1^st^ Qu.: | 3rd Qu.: |
| --- | --- | --- | --- | --- | --- |
| 29706 | 74885 | 79273 | 220139 | 57310 | 91556 |

Figure S1 Histogram of sequence reads across all samples (n=92)


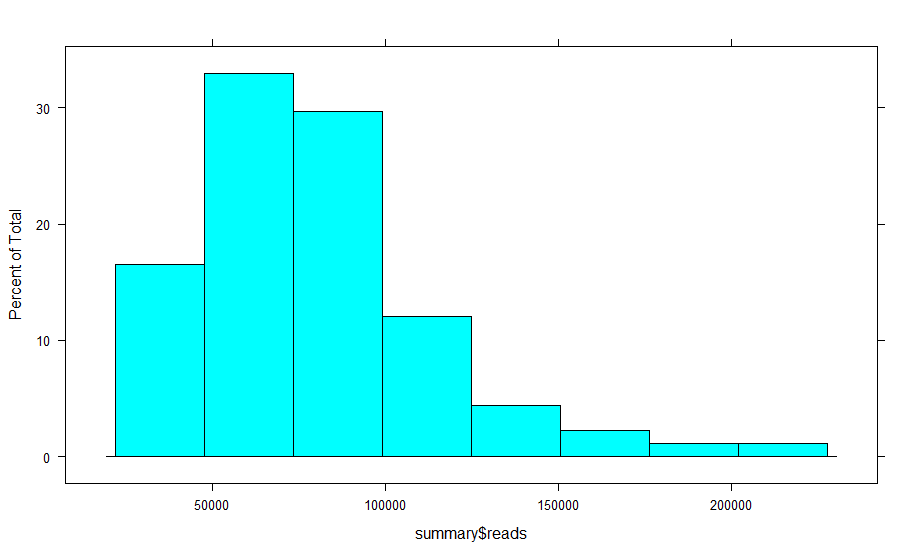


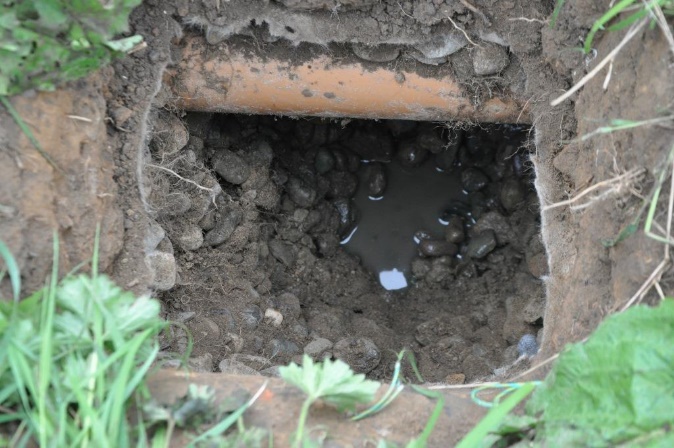

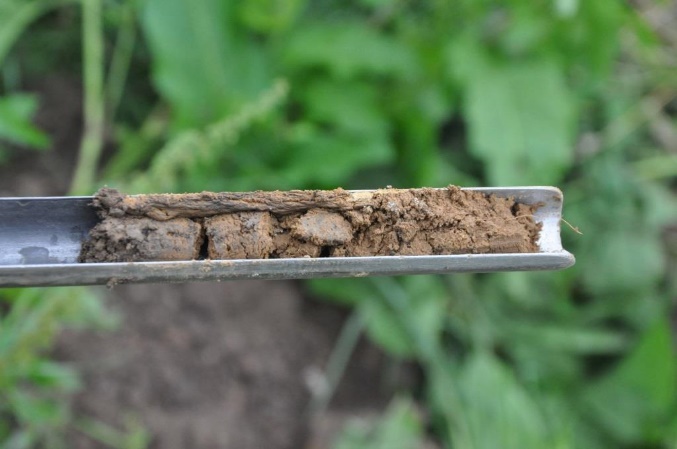

**Figure S2.** (left) Accessing STU biomat, gravel matrix and percolation pipe visible (right) and sampling using corer
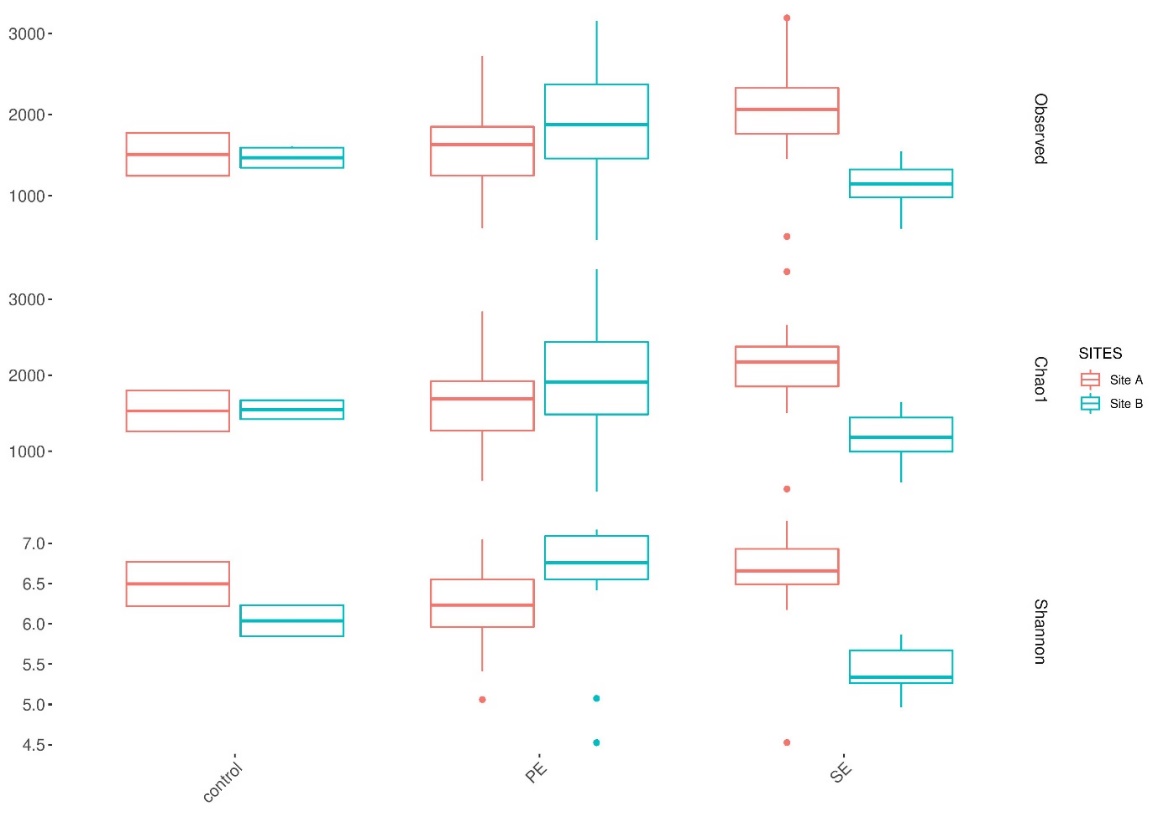


Figure S3**.** Boxplots displaying rarefied data for observed OTUs, species richness calculated using an abundance-based coverage estimates (Chao1) and alpha diversity (Shannon). Samples were aggregated on the bases of sites.

| Control | | PE | | SE | |
| --- | --- | --- | --- | --- | --- |
| Shannon | Chao1 | Shannon | Chao1 | Shannon | Chao1 |
| ns | ns | ** | * | **** | **** |

Table S2 displays the Wilcoxon test values for intersite analysis (ns Pr > 0.05, * Pr ≤ 0.05,**Pr ≤ 0.01, ***Pr ≤ 0.001 and **** Pr ≤ 0.0001) values for comparative intra-site analysis. Samples were aggregated on the bases of systems primary, secondary effluent (PE, SE)

Table S3. Mean relative abundance of key phyla within site A and site B, aggregated on the bases of systems primary, secondary effluent (PE, SE), STU top (TT), STU base (TB), control top (CT) and base (CB).

|  | **Site** | **System** | **Acidobacteriota** | **Actinobacteriota** | **Bacteroidota** | **Campylobacterota** | **Chloroflexi** |
| --- | --- | --- | --- | --- | --- | --- | --- |
| **Control** | **A** | **CB** | 17.08 ± 0.17 | 14.62 ± 4.47 | 3.64 ± 4.08 | 0 ± 0.01 | 11.16 ± 7.77 |
|  | **B** | **CB** | 27.35 ± 4.1 | 8.05 ± 0.15 | 0.36 ± 0.09 | 0 ± 0 | 9.2 ± 0.04 |
|  | **A** | **CT** | 21.05 ± 1.06 | 10.23 ± 1.37 | 3.27 ± 0.44 | 0 ± 0 | 8.07 ± 1.14 |
|  | **B** | **CT** | 20.02 ± 2.42 | 11.04 ± 2.21 | 1.98 ± 1.76 | 0 ± 0 | 8.1 ± 1.08 |
| **Primary Effluent** | **A** | **PE** | 0.24 ± 0 | 1.98 ± 0 | 16.48 ± 0 | 9.42 ± 0 | 0.29 ± 0 |
|  | **B** | **PE** | 1.3 ± 0 | 8.94 ± 0 | 4.78 ± 0 | 0.93 ± 0 | 1.2 ± 0 |
|  | **A** | **PE-TT** | 22.03 ± 5.84 | 12.58 ± 6.41 | 2.91 ± 1.66 | 0 ± 0 | 8.94 ± 1.51 |
|  | **B** | **PE-TT** | 17.27 ± 2.53 | 10.67 ± 1.45 | 1.92 ± 0.7 | 0 ± 0 | 10.32 ± 5.08 |
|  | **A** | **PE-TB(p)** | 10.22 ± 5.43 | 11.72 ± 1.13 | 7.3 ± 2.98 | 1.91 ± 2.17 | 7.67 ± 5.61 |
|  | **B** | **PE-TB(p)** | 14.01 ± 0.97 | 8.25 ± 2.7 | 1.31 ± 0.99 | 0.01 ± 0.01 | 15.84 ± 3.95 |
|  | **A** | **PE-TB(m)** | 14.48 ± 3.27 | 9.25 ± 1.58 | 2.1 ± 1.85 | 0.04 ± 0.07 | 12.43 ± 1.88 |
|  | **B** | **PE-TB(m)** | 14.11 ± 2.27 | 8.86 ± 2.19 | 2.54 ± 0.7 | 0.01 ± 0.01 | 11.2 ± 1.75 |
|  | **A** | **PE-TB(d)** | 14.62 ± 2.37 | 8.81 ± 2.25 | 2.04 ± 1.3 | 0.01 ± 0.01 | 12.82 ± 4.4 |
|  | **B** | **PE-TB(d)** | 13.39 ± 1.95 | 12.34 ± 2.64 | 1.68 ± 1.13 | 0 ± 0 | 11.46 ± 2.77 |
| **Secondary Effluent** | **A** | **SE** | 0.52 ± 0 | 1.11 ± 0 | 20.44 ± 0 | 17.56 ± 0 | 0.04 ± 0 |
|  | **B** | **SE** | 8.96 ± 6.59 | 8.36 ± 4.61 | 9.29 ± 7.03 | 0 ± 0 | 18.22 ± 1.7 |
|  | **A** | **SE-TT** | 17.89 ± 0.69 | 11.14 ± 2.01 | 4.85 ± 1.94 | 0.02 ± 0.05 | 8.48 ± 1.51 |
|  | **B** | **SE-TT** | 15 ± 7.89 | 15.71 ± 9.41 | 3.9 ± 2.28 | 0.01 ± 0.01 | 9.02 ± 4.19 |
|  | **A** | **SE-TB(p)** | 12.52 ± 1.83 | 8.64 ± 1.02 | 2.58 ± 0.68 | 0.01 ± 0.01 | 14.06 ± 2.81 |
|  | **B** | **SE-TB(p)** | 1.69 ± 0.3 | 7.26 ± 1.1 | 8.22 ± 1.79 | 2.05 ± 0.21 | 1.31 ± 0.2 |
|  | **A** | **SE-TB(m)** | 13.72 ± 0.67 | 9.06 ± 1.46 | 2.68 ± 1.84 | 0.02 ± 0.03 | 13.89 ± 2.36 |
|  | **B** | **SE-TB(m)** | 3.51 ± 0.68 | 5.94 ± 0.24 | 8.99 ± 0.85 | 2.81 ± 0.43 | 2.99 ± 1.08 |
|  | **A** | **SE-TB(d)** | 14.82 ± 2.25 | 8.33 ± 2.37 | 3.01 ± 2.13 | 0 ± 0 | 9.86 ± 0.8 |
|  | **B** | **SE-TB(d)** | 5.05 ± 3.29 | 5.06 ± 2.01 | 9.84 ± 1.56 | 0.65 ± 0.48 | 3.41 ± 1.72 |

Table S3. Mean relative abundance of key phyla within site A and site B, aggregated on the bases of systems primary, secondary effluent (PE, SE), STU top (TT), STU base (TB), control top (CT) and base (CB).

|  | **Site** | **System** | **Desulfobacterota** | **Firmicutes** | **Gemmatimonadota** | **Halobacterota** | **Latescibacterota** |
| --- | --- | --- | --- | --- | --- | --- | --- |
| **Control** | **A** | **CB** | 1.25 ± 0.76 | 6.58 ± 5.3 | 1.4 ± 1.36 | 0 ± 0 | 0.56 ± 0.23 |
|  | **B** | **CB** | 3.43 ± 0.01 | 1.63 ± 1.15 | 1.76 ± 0.19 | 0 ± 0 | 0.4 ± 0.09 |
|  | **A** | **CT** | 1.21 ± 0.21 | 2.36 ± 0.47 | 0.49 ± 0.13 | 0 ± 0 | 1.51 ± 0.03 |
|  | **B** | **CT** | 1.81 ± 0.22 | 6.39 ± 1.45 | 0.51 ± 0.17 | 0.04 ± 0.05 | 1.5 ± 0.81 |
| **Primary Effluent** | **A** | **PE** | 2 ± 0 | 27.37 ± 0 | 0.01 ± 0 | 0.1 ± 0 | 0 ± 0 |
|  | **B** | **PE** | 1.26 ± 0 | 11.28 ± 0 | 0 ± 0 | 5.01 ± 0 | 0 ± 0 |
|  | **A** | **PE-TT** | 1.16 ± 0.39 | 3.44 ± 1.68 | 0.37 ± 0.16 | 0 ± 0 | 1.26 ± 0.54 |
|  | **B** | **PE-TT** | 2.1 ± 0.72 | 5.28 ± 1.65 | 1.02 ± 0.59 | 0 ± 0.01 | 1.54 ± 0.4 |
|  | **A** | **PE-TB(p)** | 6.59 ± 6.52 | 8.85 ± 6.48 | 1.42 ± 1.1 | 0.12 ± 0.17 | 0.14 ± 0.09 |
|  | **B** | **PE-TB(p)** | 1.13 ± 0.26 | 2.16 ± 1.2 | 1.58 ± 0.39 | 0.01 ± 0.01 | 1.2 ± 0.21 |
|  | **A** | **PE-TB(m)** | 1.34 ± 0.18 | 6.85 ± 4.8 | 2.19 ± 1.17 | 0 ± 0 | 0.73 ± 0.13 |
|  | **B** | **PE-TB(m)** | 1.38 ± 0.28 | 5.01 ± 1.47 | 1.19 ± 0.19 | 0 ± 0.01 | 1.17 ± 0.11 |
|  | **A** | **PE-TB(d)** | 1.65 ± 0.93 | 3.11 ± 1.79 | 1.38 ± 0.66 | 0 ± 0 | 0.81 ± 0.52 |
|  | **B** | **PE-TB(d)** | 1.48 ± 0.29 | 7.16 ± 3.16 | 1.26 ± 0.17 | 0.01 ± 0.01 | 1.45 ± 0.22 |
| **Secondary Effluent** | **A** | **SE** | 2.33 ± 0 | 17.71 ± 0 | 0 ± 0 | 0.09 ± 0 | 0 ± 0 |
|  | **B** | **SE** | 0.68 ± 0.43 | 1.07 ± 0.33 | 0.42 ± 0.45 | 0.02 ± 0.01 | 0.11 ± 0.16 |
|  | **A** | **SE-TT** | 1.37 ± 0.3 | 5.74 ± 2.13 | 0.87 ± 0.41 | 0 ± 0 | 1.23 ± 0.22 |
|  | **B** | **SE-TT** | 1.53 ± 0.47 | 10.47 ± 8.94 | 0.69 ± 0.29 | 0.01 ± 0.01 | 1.32 ± 0.49 |
|  | **A** | **SE-TB(p)** | 1.19 ± 0.07 | 3.54 ± 0.75 | 1.63 ± 0.28 | 0 ± 0 | 1.07 ± 0.18 |
|  | **B** | **SE-TB(p)** | 2.9 ± 0.59 | 14.77 ± 2.43 | 0.02 ± 0.01 | 5.5 ± 0.9 | 0.04 ± 0.03 |
|  | **A** | **SE-TB(m)** | 1.34 ± 0.31 | 4.1 ± 0.66 | 1.87 ± 0.48 | 0 ± 0 | 1.32 ± 0.33 |
|  | **B** | **SE-TB(m)** | 4.27 ± 0.39 | 18.95 ± 2.55 | 0.07 ± 0.05 | 4.87 ± 0.75 | 0.16 ± 0.02 |
|  | **A** | **SE-TB(d)** | 1.62 ± 0.31 | 11.47 ± 3.77 | 2.42 ± 1.35 | 0 ± 0 | 0.75 ± 0.5 |
|  | **B** | **SE-TB(d)** | 8.16 ± 5.15 | 19.89 ± 4.49 | 0.24 ± 0.09 | 12.94 ± 10.61 | 0.12 ± 0.01 |

|  | **Site** | **System** | **Myxococcota** | **Planctomycetota** | **Proteobacteria** | **Synergistota** | **Verrucomicrobiota** |
| --- | --- | --- | --- | --- | --- | --- | --- |
| **Control** | **A** | **CB** | 1.5 ± 0.51 | 8.51 ± 1.32 | 14.24 ± 6.46 | 0 ± 0 | 8.07 ± 5.18 |
|  | **B** | **CB** | 1.43 ± 0.14 | 8.34 ± 0.31 | 9.77 ± 0.41 | 0 ± 0 | 6.62 ± 0.97 |
|  | **A** | **CT** | 1.23 ± 0.11 | 15.15 ± 0.49 | 13.59 ± 2.97 | 0 ± 0 | 11.87 ± 1.45 |
|  | **B** | **CT** | 1.17 ± 0.39 | 9.96 ± 0.97 | 10.91 ± 3.19 | 0 ± 0 | 14.72 ± 3.71 |
| **Primary Effluent** | **A** | **PE** | 0.1 ± 0 | 0.39 ± 0 | 21.1 ± 0 | 12.21 ± 0 | 1.02 ± 0 |
|  | **B** | **PE** | 0.13 ± 0 | 2.61 ± 0 | 46.36 ± 0 | 9.92 ± 0 | 0.8 ± 0 |
|  | **A** | **PE-TT** | 1.26 ± 0.54 | 12.84 ± 2.83 | 12.24 ± 4.1 | 0 ± 0 | 12.93 ± 3.09 |
|  | **B** | **PE-TT** | 1.13 ± 0.28 | 11.2 ± 1.23 | 11.74 ± 1.06 | 0 ± 0 | 11.81 ± 4.86 |
|  | **A** | **PE-TB(p)** | 0.87 ± 0.71 | 6.92 ± 2.29 | 25.13 ± 4.02 | 0.49 ± 0.4 | 3.21 ± 1.62 |
|  | **B** | **PE-TB(p)** | 0.6 ± 0.12 | 12.4 ± 2.08 | 13.22 ± 2.85 | 0.01 ± 0.02 | 4.99 ± 3 |
|  | **A** | **PE-TB(m)** | 1.02 ± 0.12 | 7.6 ± 1.26 | 16.3 ± 7.8 | 0 ± 0 | 3.72 ± 1.01 |
|  | **B** | **PE-TB(m)** | 1.03 ± 0.45 | 13.54 ± 2.81 | 14.69 ± 1.95 | 0 ± 0 | 7.77 ± 1.67 |
|  | **A** | **PE-TB(d)** | 0.97 ± 0.26 | 9.6 ± 1.07 | 17.07 ± 8.44 | 0 ± 0 | 3.33 ± 1.43 |
|  | **B** | **PE-TB(d)** | 1.35 ± 0.49 | 10.93 ± 2.6 | 10.68 ± 3.66 | 0 ± 0 | 6.16 ± 1.08 |
| **Secondary Effluent** | **A** | **SE** | 0.04 ± 0 | 0.27 ± 0 | 19.12 ± 0 | 12.56 ± 0 | 0.84 ± 0 |
|  | **B** | **SE** | 0.79 ± 0.12 | 15.19 ± 5.14 | 26.18 ± 5.95 | 0.02 ± 0.01 | 2.47 ± 0.47 |
|  | **A** | **SE-TT** | 1.39 ± 0.41 | 12.31 ± 1.36 | 14.71 ± 1.49 | 0 ± 0.01 | 8.9 ± 1.9 |
|  | **B** | **SE-TT** | 1.47 ± 0.66 | 8.76 ± 4.4 | 13.17 ± 5 | 0 ± 0 | 9.5 ± 4.27 |
|  | **A** | **SE-TB(p)** | 1.59 ± 0.17 | 11.37 ± 1.06 | 18.32 ± 1.71 | 0.01 ± 0.01 | 4.9 ± 1.6 |
|  | **B** | **SE-TB(p)** | 0.1 ± 0.04 | 1.51 ± 0.29 | 39.29 ± 1.75 | 8.59 ± 2.99 | 0.85 ± 0.2 |
|  | **A** | **SE-TB(m)** | 1.42 ± 0.66 | 10.51 ± 1.45 | 16.8 ± 3.96 | 0 ± 0 | 3.15 ± 1.1 |
|  | **B** | **SE-TB(m)** | 1.94 ± 1.28 | 1.52 ± 0.22 | 24.19 ± 1.39 | 9.32 ± 1.73 | 1.9 ± 0.12 |
|  | **A** | **SE-TB(d)** | 1.18 ± 0.25 | 8.82 ± 2.61 | 12.35 ± 2.63 | 0 ± 0.01 | 4.12 ± 1.9 |
|  | **B** | **SE-TB(d)** | 6.67 ± 5.96 | 3.69 ± 1.21 | 6.47 ± 2.4 | 6.81 ± 2.47 | 1.7 ± 0.28 |

Table S3. Relative abundance of key phyla within site A and site B, aggregated on the bases of systems primary, secondary effluent (PE, SE), STU top (TT), STU base (TB), control top (CT) and base (CB).

Table S4. Mean relative abundance of key genus within site A and site B, aggregated on the bases of systems primary, secondary effluent (PE, SE), STU top (TT), STU base (TB), control top (CT) and base (CB).

|  | **Site** | **System** | **Acidobacteriota; midas_g_1291** | **Acidobacteriota; midas_g_30578** | **Actinobacteriota; Gaiella** | **Actinobacteriota; Mycobacterium** | **Chloroflexi; midas_g_3277** |
| --- | --- | --- | --- | --- | --- | --- | --- |
| **Control** | **A** | **CB** | 3.6 ± 0.54 | 2.97 ± 0.64 | 0.85 ± 0.41 | 1.15 ± 0.77 | 0.97 ± 0.26 |
|  | **B** | **CB** | 2.7 ± 0.42 | 1.17 ± 0.08 | 0.51 ± 0.16 | 0.34 ± 0.04 | 0.49 ± 0.04 |
|  | **A** | **CT** | 5.95 ± 0.9 | 1.99 ± 0.33 | 0.86 ± 0.23 | 1.1 ± 0.28 | 1.16 ± 0.14 |
|  | **B** | **CT** | 5.41 ± 0.6 | 3.12 ± 1.48 | 1.28 ± 0.06 | 1 ± 0.6 | 1.04 ± 0.18 |
| **Primary** | **A** | **PE** | 0.05 ± 0 | 0 ± 0 | 0 ± 0 | 0.04 ± 0 | 0.06 ± 0 |
| **EFfluent** | **B** | **PE** | 0.05 ± 0 | 0 ± 0 | 0.03 ± 0 | 0.61 ± 0 | 0.04 ± 0 |
|  | **A** | **PE-TT** | 6.76 ± 2.45 | 2.06 ± 0.66 | 1.11 ± 0.5 | 1.42 ± 0.46 | 1.48 ± 0.26 |
|  | **B** | **PE-TT** | 4.21 ± 0.99 | 1.63 ± 0.42 | 1.19 ± 0.21 | 0.64 ± 0.32 | 1.23 ± 0.47 |
|  | **A** | **PE-TB(d)** | 2.82 ± 1.56 | 1.5 ± 0.98 | 0.45 ± 0.43 | 0.38 ± 0.22 | 1.41 ± 0.35 |
|  | **B** | **PE-TB(d)** | 2.92 ± 0.21 | 1.96 ± 0.61 | 1.14 ± 0.35 | 0.82 ± 0.42 | 1.21 ± 0.3 |
|  | **A** | **PE-TB(m)** | 3.09 ± 0.62 | 1.52 ± 0.4 | 0.35 ± 0.24 | 0.45 ± 0.18 | 1.55 ± 0.5 |
|  | **B** | **PE-TB(m)** | 2.92 ± 0.46 | 1.79 ± 0.48 | 0.8 ± 0.28 | 0.54 ± 0.28 | 0.88 ± 0.14 |
|  | **A** | **PE-TB(p)** | 0.79 ± 0.74 | 0.33 ± 0.36 | 0.15 ± 0.07 | 2.93 ± 0.94 | 0.63 ± 0.49 |
|  | **B** | **PE-TB(p)** | 3.1 ± 0.47 | 1.84 ± 0.52 | 0.72 ± 0.3 | 0.21 ± 0.12 | 1 ± 0.26 |
| **Secondary** | **A** | **SE** | 0 ± 0 | 0.02 ± 0 | 0 ± 0 | 0.54 ± 0 | 0 ± 0 |
| **Effluent** | **B** | **SE** | 0.73 ± 1 | 0.07 ± 0.1 | 0.06 ± 0.08 | 0.51 ± 0.15 | 0.24 ± 0.18 |
|  | **A** | **SE-TT** | 4.93 ± 0.54 | 2.37 ± 0.49 | 0.94 ± 0.23 | 1.11 ± 0.45 | 1.13 ± 0.36 |
|  | **B** | **SE-TT** | 3.21 ± 1.78 | 2.29 ± 1.5 | 1.47 ± 1.01 | 1.12 ± 0.54 | 0.98 ± 0.42 |
|  | **A** | **SE-TB(p)** | 3.29 ± 0.7 | 1.94 ± 0.51 | 0.3 ± 0.24 | 0.78 ± 0.12 | 1.1 ± 0.22 |
|  | **B** | **SE-TB(p)** | 0.03 ± 0.01 | 0.02 ± 0.01 | 0.06 ± 0.02 | 0.39 ± 0.06 | 0.04 ± 0.03 |
|  | **A** | **SE-TB(m)** | 3.29 ± 0.4 | 1.59 ± 0.52 | 0.34 ± 0.2 | 0.52 ± 0.18 | 1.78 ± 0.8 |
|  | **B** | **SE-TB(m)** | 0.3 ± 0.08 | 0.13 ± 0.03 | 0.33 ± 0.11 | 0.32 ± 0.03 | 0.29 ± 0.09 |
|  | **A** | **SE-TB(d)** | 3.09 ± 1.06 | 1.55 ± 0.71 | 0.56 ± 0.32 | 0.57 ± 0.14 | 0.97 ± 0.24 |
|  | **B** | **SE-TB(d)** | 0.38 ± 0.3 | 0.03 ± 0.03 | 0.21 ± 0.05 | 0.06 ± 0.06 | 0.29 ± 0.23 |

Table S4. Relative abundance of key genus within site A and site B, aggregated on the bases of systems primary, secondary effluent (PE, SE), STU top (TT), STU base (TB), control top (CT) and base (CB).

|  | **Site** | **System** | **Chloroflexi; midas_g_6161** | **Firmicutes; Bacillus** | **Firmicutes; Clostridium_sensu_stricto_1** | **Firmicutes; o__Bacillales_OTU_1** | **Firmicutes; Romboutsia** |
| --- | --- | --- | --- | --- | --- | --- | --- |
| **Control** | **A** | **CB** | 2.02 ± 2.08 | 0.59 ± 0.03 | 0.02 ± 0.01 | 2.77 ± 2.51 | 0.01 ± 0.02 |
|  | **B** | **CB** | 1.29 ± 0.09 | 0.28 ± 0.29 | 0.03 ± 0.02 | 0.28 ± 0.27 | 0 ± 0 |
|  | **A** | **CT** | 0.81 ± 0.07 | 0.51 ± 0.1 | 0 ± 0 | 0.86 ± 0.2 | 0.01 ± 0.01 |
|  | **B** | **CT** | 0.7 ± 0.2 | 1.86 ± 0.43 | 0.07 ± 0.06 | 2.03 ± 0.98 | 0.05 ± 0.03 |
| **Primary** | **A** | **PE** | 0 ± 0 | 0.17 ± 0 | 0.91 ± 0 | 0 ± 0 | 0.55 ± 0 |
| **Effluent** | **B** | **PE** | 0 ± 0 | 0.16 ± 0 | 2.75 ± 0 | 0.11 ± 0 | 3.33 ± 0 |
|  | **A** | **PE-TT** | 0.92 ± 0.32 | 0.56 ± 0.24 | 0.04 ± 0.03 | 1.43 ± 0.66 | 0 ± 0.01 |
|  | **B** | **PE-TT** | 1.38 ± 0.85 | 1.18 ± 0.41 | 0.03 ± 0.01 | 1.86 ± 0.93 | 0.03 ± 0.02 |
|  | **A** | **PE-TB(d)** | 1.24 ± 0.42 | 0.33 ± 0.38 | 0.05 ± 0.04 | 0.7 ± 0.51 | 0.06 ± 0.06 |
|  | **B** | **PE-TB(d)** | 0.46 ± 0.11 | 1.58 ± 0.92 | 0.14 ± 0.1 | 1.8 ± 0.82 | 0.08 ± 0.05 |
|  | **A** | **PE-TB(m)** | 1.12 ± 0.43 | 0.5 ± 0.38 | 0.06 ± 0.06 | 1.93 ± 2.08 | 0.07 ± 0.08 |
|  | **B** | **PE-TB(m)** | 0.4 ± 0.08 | 0.69 ± 0.4 | 0.1 ± 0.05 | 1.98 ± 0.5 | 0.11 ± 0.03 |
|  | **A** | **PE-TB(p)** | 0.71 ± 0.77 | 0.51 ± 0.39 | 0.32 ± 0.28 | 1.16 ± 1.29 | 0.18 ± 0.11 |
|  | **B** | **PE-TB(p)** | 0.32 ± 0.13 | 0.34 ± 0.34 |  | 0.54 ± 0.31 | 0.11 ± 0.04 |
| **Secondary** | **A** | **SE** | 0 ± 0 | 0.22 ± 0 | 0.2 ± 0 | 0 ± 0 | 0.11 ± 0 |
| **Effluent** | **B** | **SE** | 0 ± 0 | 0.03 ± 0.04 | 0.35 ± 0.13 | 0 ± 0 | 0.29 ± 0.03 |
|  | **A** | **SE-TT** | 0.82 ± 0.17 | 0.9 ± 0.29 | 0.05 ± 0.1 | 1.42 ± 0.41 | 0.02 ± 0.01 |
|  | **B** | **SE-TT** | 0.78 ± 0.42 | 2.52 ± 2.64 | 0.15 ± 0.09 | 2.83 ± 2.39 | 0.23 ± 0.27 |
|  | **A** | **SE-TB(p)** | 1.19 ± 0.46 | 0.45 ± 0.14 | 0.06 ± 0.01 | 0.9 ± 0.32 | 0.05 ± 0.02 |
|  | **B** | **SE-TB(p)** | 0.01 ± 0.01 | 0.37 ± 0.11 | 3.3 ± 0.59 | 0.41 ± 0.15 | 4.97 ± 1.01 |
|  | **A** | **SE-TB(m)** | 1.63 ± 0.28 | 0.45 ± 0.16 | 0.06 ± 0.03 | 0.86 ± 0.25 | 0.06 ± 0.02 |
|  | **B** | **SE-TB(m)** | 0.06 ± 0.02 | 0.8 ± 0.21 | 4.89 ± 1.01 | 0.93 ± 0.08 | 6.97 ± 1.68 |
|  | **A** | **SE-TB(d)** | 0.52 ± 0.19 | 0.48 ± 0.31 | 0.39 ± 0.63 | 3.18 ± 1.67 | 0.03 ± 0.01 |
|  | **B** | **SE-TB(d)** | 0.02 ± 0.03 | 0.77 ± 0.2 | 1.71 ± 1.11 | 0.99 ± 0.28 | 4.36 ± 3.16 |

Table S4. Relative abundance of key genus within site A and site B, aggregated on the bases of systems primary, secondary effluent (PE, SE), STU top (TT), STU base (TB), control top (CT) and base (CB).

|  | **Site** | **System** | **Halobacterota; Methanosarcina** | **Latescibacterota; midas_g_2686** | **Nitrospirota; Nitrospira** | **Planctomycetota; Pirellula** | **Proteobacteria; Thauera** |
| --- | --- | --- | --- | --- | --- | --- | --- |
| **Control** | **A** | **CB** | 0 ± 0 | 0.43 ± 0.26 | 0.5 ± 0.43 | 0.76 ± 0.24 | 0 ± 0 |
|  | **B** | **CB** | 0 ± 0 | 0.29 ± 0.09 | 0.75 ± 0.02 | 0.44 ± 0.11 | 0 ± 0 |
|  | **A** | **CT** | 0 ± 0 | 1.14 ± 0.08 | 0.34 ± 0.04 | 1.36 ± 0.55 | 0 ± 0 |
|  | **B** | **CT** | 0.03 ± 0.03 | 1.21 ± 0.83 | 0.33 ± 0.09 | 0.81 ± 0.16 | 0 ± 0 |
| **Primary** | **A** | **PE** | 0.03 ± 0 | 0 ± 0 | 0.07 ± 0 | 0 ± 0 | 0 ± 0 |
| **Effluent** | **B** | **PE** | 3.1 ± 0 | 0 ± 0 | 0.05 ± 0 | 0.25 ± 0 | 5.98 ± 0 |
|  | **A** | **PE-TT** | 0 ± 0 | 1.09 ± 0.47 | 0.18 ± 0.07 | 0.71 ± 0.3 | 0 ± 0 |
|  | **B** | **PE-TT** | 0 ± 0.01 | 1.33 ± 0.36 | 0.29 ± 0.11 | 1.03 ± 0.35 | 0 ± 0 |
|  | **A** | **PE-TB(d)** | 0 ± 0 | 0.67 ± 0.48 | 0.54 ± 0.14 | 1.2 ± 0.52 | 0.09 ± 0.1 |
|  | **B** | **PE-TB(d)** | 0.01 ± 0.01 | 1.22 ± 0.23 | 0.86 ± 0.3 | 0.76 ± 0.2 | 0.02 ± 0.01 |
|  | **A** | **PE-TB(m)** | 0 ± 0 | 0.57 ± 0.11 | 1.09 ± 0.5 | 0.66 ± 0.3 | 0.18 ± 0.21 |
|  | **B** | **PE-TB(m)** | 0 ± 0.01 | 0.86 ± 0.09 | 1.02 ± 0.34 | 1.42 ± 0.25 | 0.04 ± 0.02 |
|  | **A** | **PE-TB(p)** | 0.04 ± 0.06 | 0.11 ± 0.09 | 0.09 ± 0.08 | 0.44 ± 0.28 | 0.38 ± 0.3 |
|  | **B** | **PE-TB(p)** | 0.01 ± 0.01 | 0.96 ± 0.2 | 1.17 ± 0.18 | 1.27 ± 0.2 | 0.05 ± 0.03 |
| **Secondary** | **A** | **SE** | 0 ± 0 | 0 ± 0 | 0 ± 0 | 0.04 ± 0 | 0.02 ± 0 |
| **Effluent** | **B** | **SE** | 0 ± 0 | 0.11 ± 0.16 | 0.3 ± 0.4 | 1.13 ± 0.64 | 0 ± 0 |
|  | **A** | **SE-TT** | 0 ± 0 | 0.88 ± 0.17 | 0.43 ± 0.14 | 1.06 ± 0.25 | 0 ± 0 |
|  | **B** | **SE-TT** | 0 ± 0.01 | 1.06 ± 0.43 | 0.37 ± 0.21 | 1.08 ± 0.7 | 0 ± 0 |
|  | **A** | **SE-TB(p)** | 0 ± 0 | 0.8 ± 0.13 | 1.37 ± 0.2 | 1.08 ± 0.3 | 0 ± 0.01 |
|  | **B** | **SE-TB(p)** | 3.33 ± 0.19 | 0.01 ± 0.01 | 0.02 ± 0.01 | 0.15 ± 0.06 | 9.21 ± 1.05 |
|  | **A** | **SE-TB(m)** | 0 ± 0 | 1 ± 0.31 | 1.09 ± 0.22 | 1.08 ± 0.27 | 0.17 ± 0.18 |
|  | **B** | **SE-TB(m)** | 2.6 ± 0.25 | 0.09 ± 0.02 | 0.09 ± 0.01 | 0.15 ± 0.08 | 1.49 ± 0.23 |
|  | **A** | **SE-TB(d)** | 0 ± 0 | 0.57 ± 0.34 | 1.18 ± 0.49 | 0.91 ± 0.42 | 0 ± 0 |
|  | **B** | **SE-TB(d)** | 10.3 ± 8.85 | 0.03 ± 0.03 | 0.12 ± 0.11 | 0.05 ± 0.05 | 0.31 ± 0.25 |

.


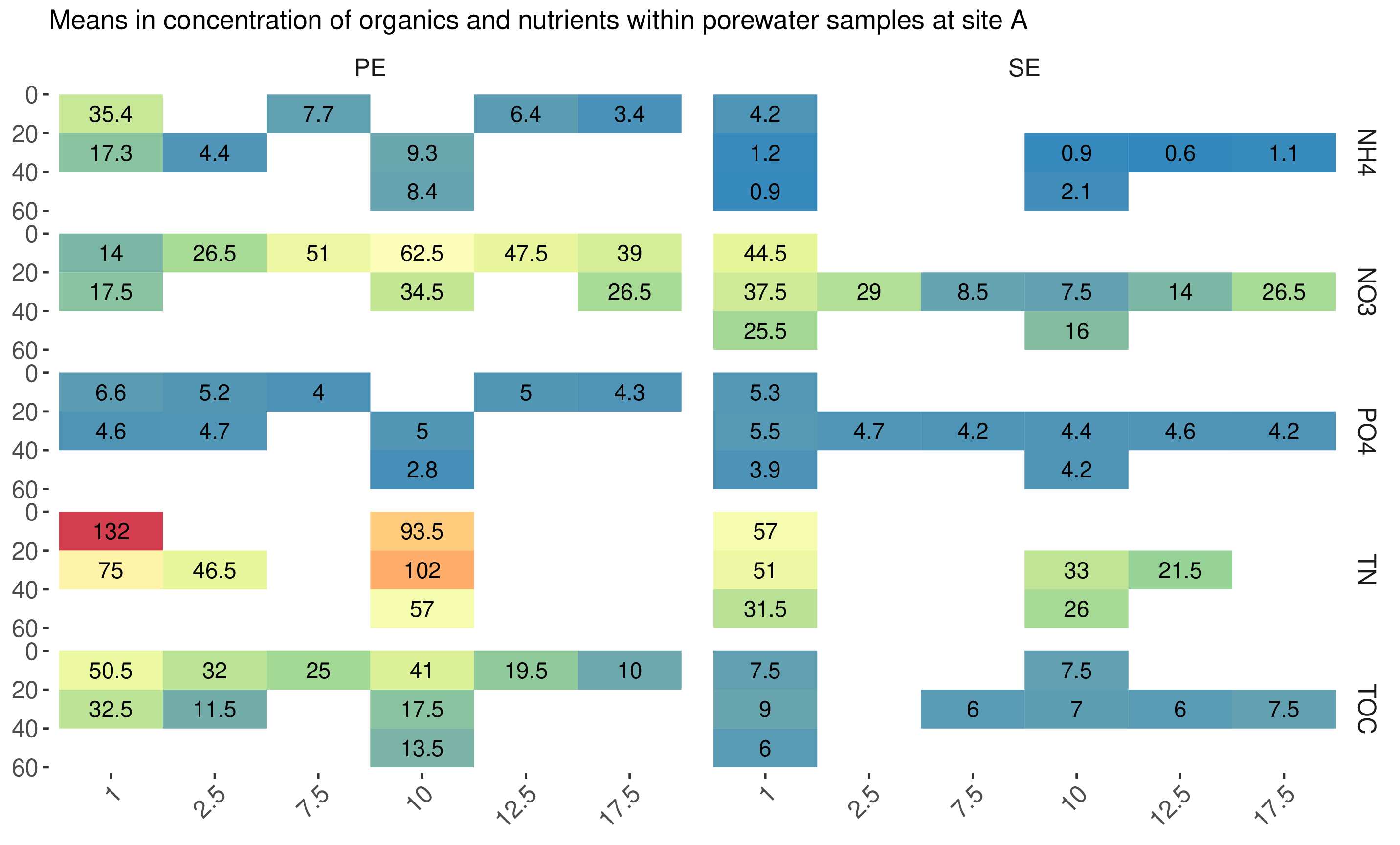


**Figure S4** Means in concentration of ammonium, nitrate, ortho-phosphate, total nitrogen and total organic carbon within the porewater samples at site A, throughout the year of sampling.

**
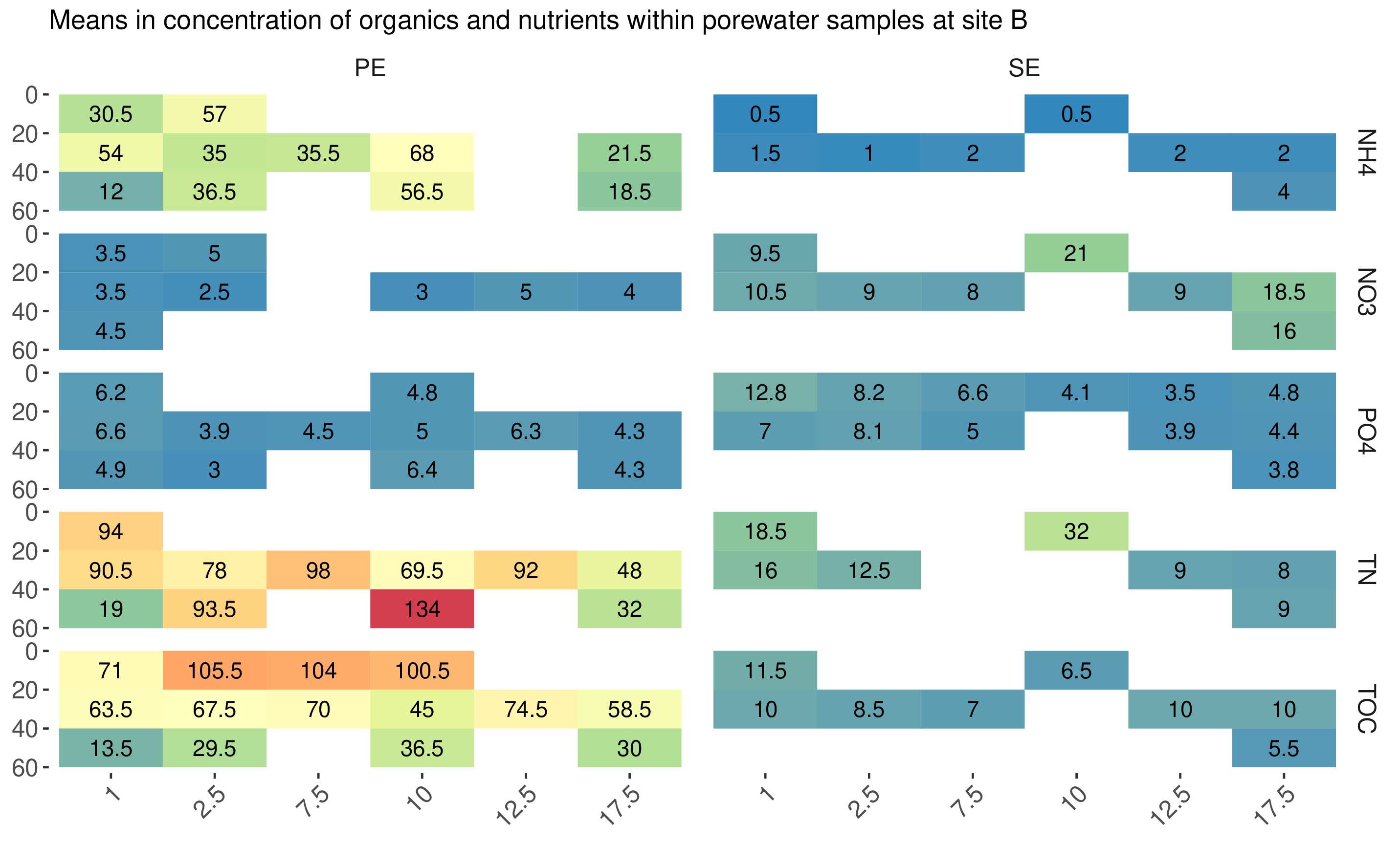
**

**Figure S5** Means in concentration of ammonium, nitrate, ortho-phosphate, total nitrogen and total organic carbon within the porewater samples at site B, throughout the year of sampling.


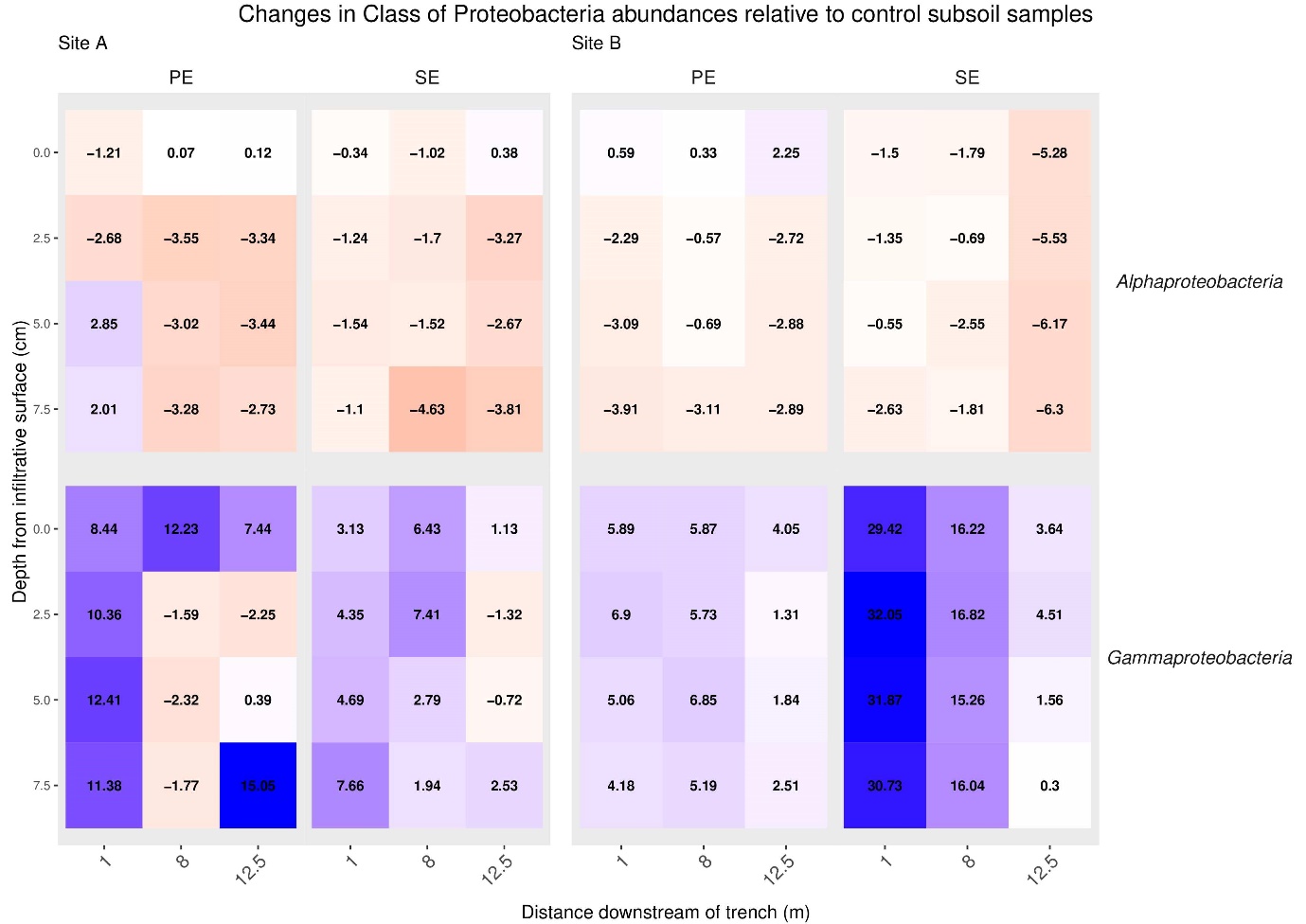


**Figure S6** changes in relative abundance of Gammaproteobacteria and Alphaproteobacteria relative to the control subsoils, increases are marked in blue and reductions in red


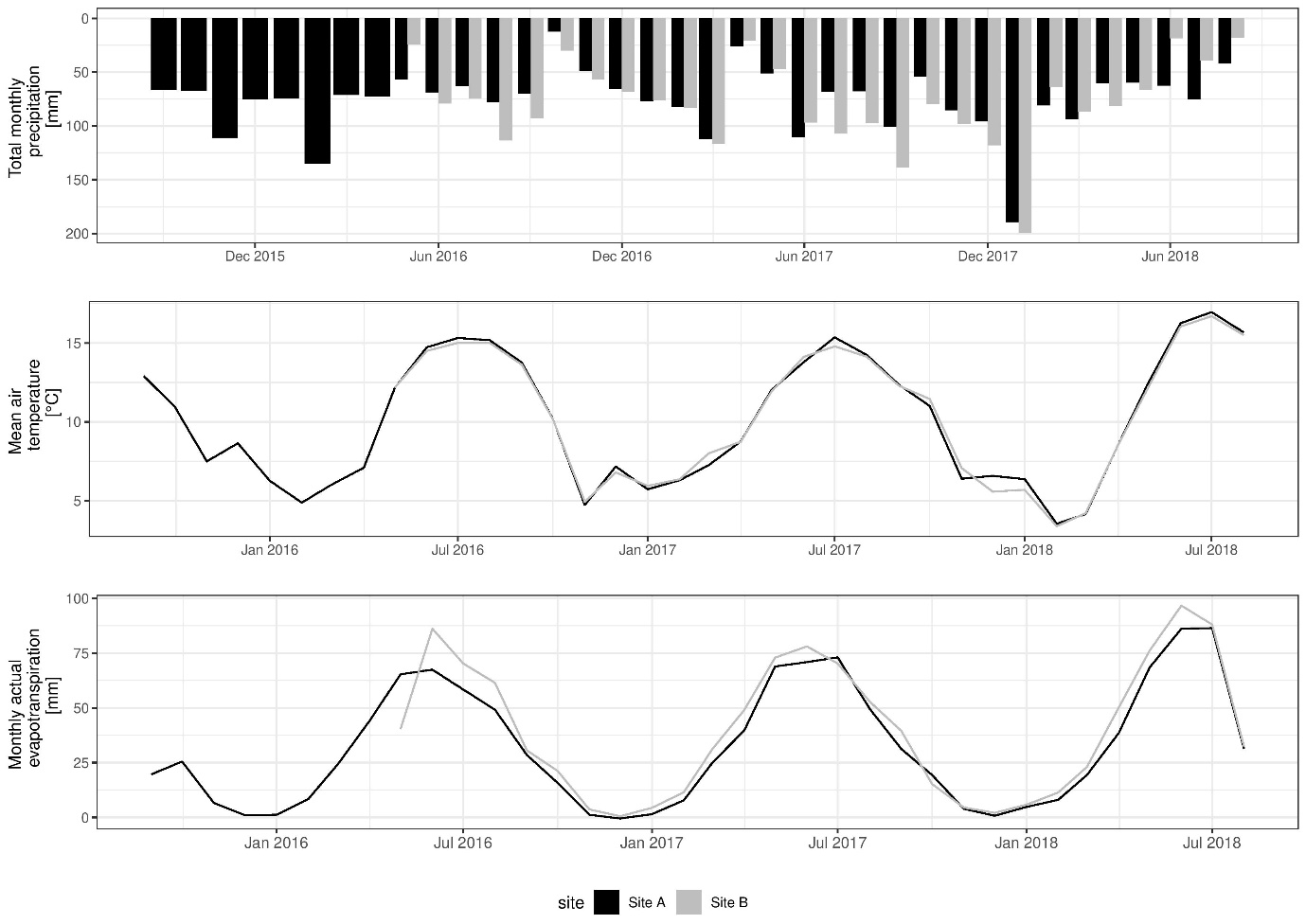


**Figure S7** Total monthly precipitation, mean monthly air temperatures and monthly actual evapotranspiration for both sites between January 2016 to August 2018.
